# *N*-Acetyl-Cysteine: Modulating the Cysteine Redox Proteome in Neurodegenerative Diseases

**DOI:** 10.3390/antiox11020416

**Published:** 2022-02-18

**Authors:** Marcos Martinez-Banaclocha

**Affiliations:** Department of Pathology, Lluis Alcanyis Hospital, Xátiva, 46800 Valencia, Spain; martinez_marben@gva.es

**Keywords:** Alzheimer, cysteine, Huntington, *N*-acetyl-cysteine, Parkinson, redox, proteome, ROS, RNS, RSS

## Abstract

In the last twenty years, significant progress in understanding the pathophysiology of age-associated neurodegenerative diseases has been made. However, the prevention and treatment of these diseases remain without clinically significant therapeutic advancement. While we still hope for some potential genetic therapeutic approaches, the current reality is far from substantial progress. With this state of the issue, emphasis should be placed on early diagnosis and prompt intervention in patients with increased risk of neurodegenerative diseases to slow down their progression, poor prognosis, and decreasing quality of life. Accordingly, it is urgent to implement interventions addressing the psychosocial and biochemical disturbances we know are central in managing the evolution of these disorders. Genomic and proteomic studies have shown the high molecular intricacy in neurodegenerative diseases, involving a broad spectrum of cellular pathways underlying disease progression. Recent investigations indicate that the dysregulation of the sensitive-cysteine proteome may be a concurrent pathogenic mechanism contributing to the pathophysiology of major neurodegenerative diseases, opening new therapeutic opportunities. Considering the incidence and prevalence of these disorders and their already significant burden in Western societies, they will become a real pandemic in the following decades. Therefore, we propose large-scale investigations, in selected groups of people over 40 years of age with decreased blood glutathione levels, comorbidities, and/or mild cognitive impairment, to evaluate supplementation of the diet with low doses of *N*-acetyl-cysteine, a promising and well-tolerated therapeutic agent suitable for long-term use.

## 1. Introduction

The significant increase in human life expectancy has resulted in an insidious rise in the prevalence of age-associated neurodegenerative diseases, becoming a true pandemic affecting most countries worldwide. It has been estimated that Alzheimer’s disease will affect nearly half of the population over 85 years of age by 2050. There is no remedial therapy for neurodegenerative diseases, and only palliative medications address different symptoms of each condition. Therefore, prevention and early diagnosis of these neurodegenerative diseases are crucial to improving patient outcomes. Though the chances of underdiagnosis and misdiagnosis will probably remain high, some diagnostic tools and imaging techniques help us to detect the brain’s activity, the extent of the injury, and the early location of deterioration in the CNS. The discovery that most neurodegenerative diseases share homeostasis deregulation of proteins and pathologic accumulation of specific proteins has prompted a search to find strategies to prevent or interfere with the disturbed proteome of each disorder.

Prevalent neurodegenerative diseases, such as Alzheimer’s disease (AD), Parkinson’s disease (PD), Huntington’s disease (HD), amyotrophic lateral sclerosis (ALS), and frontotemporal dementia (FTD) show differences in clinical presentation and evolution with characteristic alterations affecting the aggregation of specific proteins and their deposition in central and peripheral specific neural cells, which seem typical of each disease. Although the impact on brain dysfunction develops according to different rhythms, the diseases each entail protein accumulation, mitochondrial dysfunction, and oxidative damage that finally end in neuronal death.

We have previously proposed that some common neurodegenerative disorders, such as AD and PD, each involve the dysregulation of the sensitive-cysteine proteome [1,2,3,4,5]. In the present paper, we extend this proposition to other neurodegenerative diseases, suggesting that a disturbance of the redox cysteine proteome mediates all age-associated degenerative diseases. In an effort to reconcile the role played by many different etiopathogenic factors and associated conditions in neurodegenerative disorders and the progressive pathological evolution of these diseases, with or without individual genetic susceptibility, in addition to toxic environmental factors and age-associated oxidative damage, we propose a mechanism of convergent redox proteomic dysregulation that impairs the biochemical pathways involved in each specific disease.

A scientific and rational strategy to solve the growing problem of neurodegenerative diseases requires a long and aggressive international initiative using well-known and safe compounds. *N*-acetyl-cysteine (NAC) is a natural amino acid, modified to increase its bio-disponibility, that may be easily incorporated into the diet. NAC has proven beneficial effects in diabetes, hypertension, obesity, and cardiovascular disturbance management, many of which are associated with and contribute to neurodegeneration. Since the prevention of neurodegenerative diseases remains without clinically significant therapeutic advancement, the present review proposes a large-scale intervention in people over 40 years of age with decreased blood glutathione levels, comorbidities, and/or mild cognitive impairment involving supplementation of the diet with 600 mg of NAC three times a week. These interventions have the potential to slow the onset and progression of dementia, reducing the neurodegenerative disease burden.

## 2. The Sensitive-Cysteine Redox Proteome (Cysteinet)

Protein homeostasis (proteostasis) is central to maintaining the functional cellular proteome, a dynamic complex process involving response to different physiological and pathological conditions [6,7]. Proteostasis comprises synchronization from protein synthesis in the ribosome through to posttranslational modification, trafficking to each subcellular compartment, assembly into protein macro-complexes, signaling, and degradation [7,8], depending on proper protein folding and transport by chaperones [9,10]. The post-translational modification of proteins (PTMPs) increases the complexity and diversity of proteins, which are differentially expressed in distinct tissues determining the versatility and functionality of most biochemical pathways in the cell. PTMPs principally include phosphorylation, acetylation, allosteric modification, and redox regulations. Redox PTMPs affect many different amino acids, but cysteine is the most readily affected, possessing a thiol group (-SH) which is deprotonated at physiological pH, allowing reversible redox modifications. 

Redox-regulated proteins comprise the redox proteome (redoxome), in which specific cysteine residues play a crucial role through reversible redox modifications [11,12]. The number of cysteine residues in proteins comprises approximately 2% of the total, though this amino acid is favorably conserved in proteins through evolution because of its highly functional role. Since cysteine is derived from methionine, it is not considered an essential amino acid. Nevertheless, cysteine is fundamental to maintaining the synthesis, structure, and functional versatility of many proteins and small polypeptides, such as glutathione. Sensitive-cysteine residues in proteins act in diverse redox reactions, including the reversible change of the catalytic function in enzymes, such as in the mitochondrial respiratory chain. Sensitive cysteine residues in proteins also participate in structural and contractile functions supporting the cytoskeletal architecture and movements of cells, the folding and transportation of proteins via membranes, the modulation of cellular signals, the regulation of DNA synthesis and expression, and the retention and consumption of fuels [12,13]. Quantitative thiol reactivity methods have identified many redox-sensitive cysteine residues in proteins, including proteins involved in translation, histone modification, mRNA splicing, and growth regulatory pathways [12]. These findings have revealed the unexpected importance of the redox cysteine proteome in regulating vital pathways in a complex organism [12]. Notably, 54% of cysteine residues in essential proteins showed similar intrinsic reactivity in *C. elegans* and human cells. For example, the Cys33 residue in glutathione S-transferase exhibited almost the same reactivity in *C. elegans* and *Homo sapiens* [13]. In other cases, such as human glyceraldehyde 3-phosphate dehydrogenase (GAPDH), the reactive Cys158 site within all four orthologs in *C. elegans* shows the same reactivity in GPD-1 and GPD-2/3 but a higher reactivity in GPD-4, suggesting there is a correlation between the amino acid sequence and intrinsic cysteine reactivity [12]. However, subtle changes in flanking sequences can influence redox cysteine reactivity, and, therefore, the redox sensitivity of cysteine residues is modulated by their microenvironment [12].

Redox modification of sensitive-cysteine residues maintains the thiol status of critical proteins, defending the cell against reactive species and contributing to the detoxification of xenobiotics and their metabolites. Hence, cysteine is viewed as the main extracellular antioxidant, encountered principally as disulfide cystine because the extracellular conditions are rather oxidizing. This reservoir is integrated and complements the principal intracellular antioxidant glutathione (GSH), though the two are not in balance [14]. The cysteine residues of the tripeptide GSH participate in most of the complex network of enzyme-catalyzed reactions in practically all sub-cellular organelles and the cell’s cytoplasm [14]. Glutathione can reduce disulfide bridges in cytoplasmic proteins, when it is converted to glutathione disulfide (GSSG) that is reduced back through glutathione reductase utilizing NADPH as an electron donor. In addition, GSH is a cofactor for diverse antioxidant enzymes, such as glutathione reductases, glutathione peroxidases, and glutathione S-transferases, which participate in redox homeostasis [14].

The broad but specific distribution of redox-sensitive cysteines across the proteome indicates that essential physiological functions must be subject to exquisite redox regulation, highlighting the biological importance of redox-sensitive cysteine residues in vivo. The metabolism produces reactive oxygen, nitrogen, and sulfur species (ROS, RNS, and RSS, respectively) that operate as cellular redox homeostasis intermediates, regulating different metabolic processes through reversible or irreversible redox modifications of reactive cysteine residues [1,11,12,15]. Reactive species trigger radical transfer, a defensive cellular instrument against oxidative injury in proteins, in which oxidation at specific sites in the protein involves a chain of electron transfer towards further redox amino acid residues, such as cysteine or methionine, to neutralize the oxidative impairment at the initial point [16]. This mechanism works physiologically at the catalytic sites of some enzymes and among diverse enzymatic proteins, such as in the mitochondrial respiratory chain [17].

The proteasome is liable for the ubiquitin-dependent and -independent degradation of oxidatively damaged proteins. Under physiological conditions, most misfolded proteins are ubiquitylated and degraded [18]. Protein degradation in the proteasome requires unfolded and readily available domains in those proteins susceptible to degradation, which depends on successive substrate processing through to final degradation [18,19]. The initial binding of the ubiquitinated protein to the E1 ubiquitin-activating enzyme depends on a thiol ester bond [20], as is the case for transferring the activated ubiquitin to the E2 ubiquitin-conjugating enzyme via an active cysteine residue within the E2 protein [20,21]. Furthermore, ubiquitin E3 ligases contain reactive cysteine residues that modulate ubiquitylation and suffer cysteine modifications affecting the ligase activity. Indeed, the redox-regulated E3 ligase adaptor Kelch-like ECH-associated protein 1 (Keap1) has numerous reactive cysteines determining its interactions with the redox-associated transcription factor Nrf2 [22,23]. Nrf2 is a critical component of the antioxidant cell response by incorporating signals derived from misfolded protein accumulation to coordinate an adequate transcriptional response. In consequence, Nrf2 coordinates the endoplasmic reticulum (ER), the proteasome, and autophagy functionality [24,25], participating in the cellular response against oxidative and electrophilic compounds. In this regard, the ubiquitin E3 ligase Keap1 acts as a reactive cysteine redox sensor leading to Nrf2 ubiquitination and proteasomal degradation.

Moreover, Nrf2 regulates vital enzymes of the GSH metabolism in the brain, such as cystine/glutamate transport, γ-glutamate-cysteine synthetase (γ-GS), glutamate-cysteine ligase, glutathione reductase (GR), and glutathione peroxidase (GPX) [25]. Oxidative protein folding in the ER depends on the protein disulfide isomerase (PDI) and the endoplasmic oxidoreductin 1 (Ero1) sulfhydryl oxidases as disulfide donors. PDI modulates protein folding, catalyzing disulfide bonds between reactive cysteine residues due to the redox reactions of its cysteine residues. Interestingly, the oxidative modification of a single cysteine residue (Cys663) within the kinase activation loop of the ER protein, IRE-110, can suppress the unfolded protein response stimulating the p38 mitogen-activated protein kinase (MAPK) antioxidant signal simultaneously [24].

The redox cysteine proteome in *C. elegans* is expressed through different tissues and organs with sensitive-cysteine proteins widely distributed in the primary cellular organelles, including mitochondria, the endoplasmic reticulum, and the nucleus [12]. Genetic and molecular analyses of the *C. elegans* redoxome have identified many fundamental cellular processes, including the ubiquitin-proteasome pathway that showed a high representation of functional cysteine proteins. The authors suggested that the cysteine enrichment in critical metabolic processes (i.e., glycolysis, the tricarboxylic acid cycle, and the pentose phosphate pathway) is consistent with the hypothesis that the redox modification of proteins modulates metabolic fluxes to maintain cellular redox homeostasis [12]. Thus, redox regulation of sensitive-cysteines may control the ubiquitylation and the subsequent proteasomal degradation of proteins, serving as a mechanism for reactive species to sense and back-regulate practically all the metabolism [7,26]. This proteasomal cysteine redox sensitivity plays a critical role in cellular redox homeostasis, and it appears to be central to understanding neurodegeneration.

The cellular redox proteome encompasses all proteins that suffer reversible and irreversible redox reactions under physiological and pathological conditions. Cys, Met, and seleno-cysteine residues at the shell and other steric functional sites of proteins are especially susceptible to redox changes. Nevertheless, other amino acids (Trp, Tyr, and Arg) can react with derivatives from oxidative metabolism [1,15]. Therefore, the sensitive-cysteine redox proteome (cysteinet) is considered one of the functional compartments of the redox proteome. Cysteinet engages all peptides and proteins that possess functional redox cysteine residues in their structure, including the small tripeptide glutathione. This network interconnects reactive species (e.g., ROS, RNS, and RSS) with cysteine-containing proteins to control cellular survival, regeneration, and death [1]. These sensitive-cysteine-containing proteins (SCCPs) are regulated by the same thiol radical into cysteine residues, though they can have diverse metabolic, signaling, and structural functions. SCCPs function as cellular detectors, synchronizing over brief timescales distinct cellular functions under the maintenance of the redox environment. Sensitive-cysteines can suffer various redox changes (e.g., s-glutathionylation, s-nitrosylation, sulfenylation, disulfide bond formation) under particular physiological or pathological conditions, resulting in a reversible shift in protein function [1]. It has been suggested that cysteinet is crucial in neurodegeneration [1,2,3,4,5] and brain development, which is also under the control of SCCPs at very early stages [27]. Further investigations may be necessary to better understand psychiatric diseases, such as schizophrenia and bipolar disorders [28,29].

## 3. The Sensitive-Cysteine Redox Proteome (Cysteinet) in Aging

Aging is associated with the oxidative damage of macromolecules mediated by reactive species [30,31,32]. Aged healthy humans show an age-associated decline in the cysteine/cystine proportion throughout life, and reduction in the total glutathione content and GSH/GSSG ratio [33], indicating an age-related modification of redox homeostasis toward pro-oxidant states [34], affecting multiple regulatory paths that depend on the integrity of SCCPs [35,36], including mitochondrial ones [37]. Thus, aging raises the rate of irreversible injury to structural and metabolic proteins hampering normal cellular functions and homeostasis [1]. Proteomic studies show that aging is associated with a deleterious transition in redox-regulated proteins resulting in substantial shifts in the glycolytic enzymes and the regulatory enzymes handling energy metabolism in muscular post-mitotic cells [38]. Moreover, normal brain aging is characterized by deregulated expression of specific aggregation-prone proteins predisposing to Aβ and tau deposition, as well as suboptimal expression of homeostatic proteins [39]. Genome-wide gene expression investigations have supplied confirmation of decreased mitochondrial function during aging and declined gene expression implicated in mitochondrial energy metabolism in humans with cognitive deterioration and AD [40]. On the other hand, the effect of caloric restriction in aging is mediated by decrease in body temperature, the rate of metabolism, and decreased generation of reactive species balancing age-related oxidative injury and stabilizing mitochondrial activity in neurons [41]. Consequently, age-associated oxidative change of SCCPs may be the Achilles’ heel for senescence and neurodegenerative disturbances [1].

Recent studies demonstrate that cysteine reactivity profiling can complement transcriptomic and proteomic analyses in complex organisms through activity-based protein profiling. This technique identifies reactive cysteine residues in catalytic critical sites in complex proteomes [42]. Protein homeostasis is one of the essential mechanisms of cells maintaining the adequate functional proteome in each organelle and its integration in different stress-inducing conditions, including cellular aging, which is associated with a decline in proteostasis [6,7]. Proteostasis is a high-energy-dependent part of the proteome that involves different functions, such as editing, transfer, folding, and degradation of proteins across cellular organelles, including the cytosol, nucleus, mitochondria, and endoplasmic reticulum (ER) [6,7,26]. Proteins are actively synthesized in the ER, and cysteine residues supply the proper disulfide bonds to provide the 3D structure and conformational stability to the recently synthesized proteins [43]. Then, the redox environment of the ER ensures the correct folding of proteins that is exquisitely regulated by a network of oxidases and PDIs [6,7,26]. It has been shown that the redox states in the cytosol and the ER are expressed differently in aging, stress-inducing circumstances and diseases. The ER becomes more reduced while the cytosol becomes more oxidizing in reaction to these stress-inducing conditions [6,7,26].

The relationship between redox cysteine modifications and aging involves proteins from several pathways to maintain protein homeostasis and cell survival. Wide-scale proteomic approaches demonstrated the presence of cysteine redox networks with tissue-specific changes in thiol oxidation during aging, including tRNA multi-synthetase complex members [6,7]. Therefore, thiol reduction during aging suggests that redox versatility is compromised [6,7], likely contributing to age-related neurodegeneration [1].

**Figure 1:**
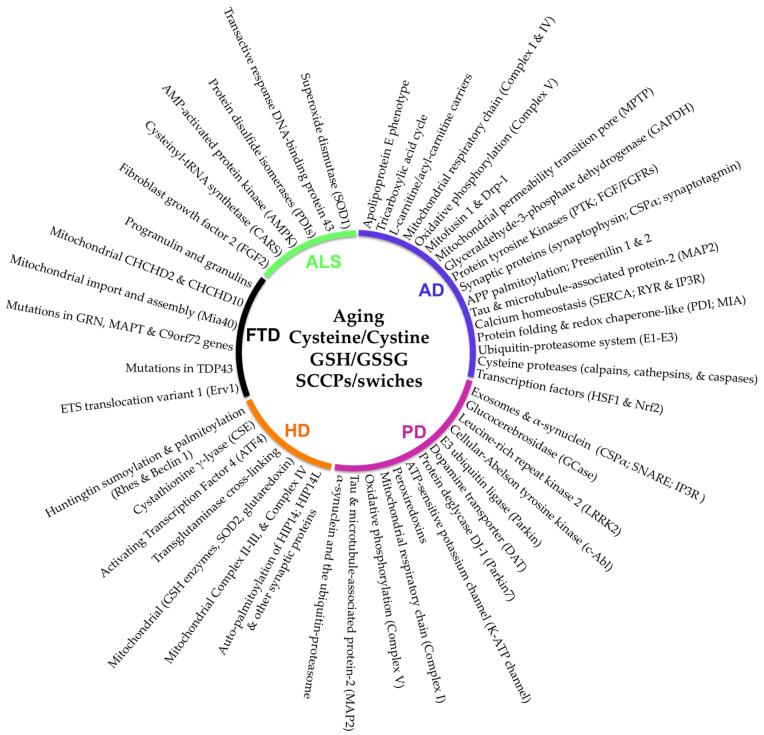
Schematic overview of cysteine redox proteome (cysteinet) in neurodegenerative diseases. An essential subset of the principal sensitive-cysteine-containing proteins (SCCPs) in Alzheimer’s disease (AD), Parkinson’s disease (PD), Huntington’s disease (HD), frontotemporal dementia (FTD), and amyotrophic lateral sclerosis (ALS) are indicated. The scheme also shows some genes associated with the development of AD, PD, HD, FTD, and ALS (see the main text). Protein misfolding and defective ubiquitin-proteasome function contribute to the pathophysiology and progression of neurodegenerative disorders. A subset of SCCPs concerns the autophagy-lysosome and mitophagy processes leading to the accumulation of proteins and the formation of protein depositions. The impairment of mitochondrial bio-energetic ability, decreased glutathione (GSH) concentrations, and reactive oxygen species (ROS) over-production can also contribute to neuronal death. A mechanism to be investigated is the function of the cysteine redox homeostasis dysregulation working via cysteine switches controlling the process of critical cellular paths.

**Figure 2:**
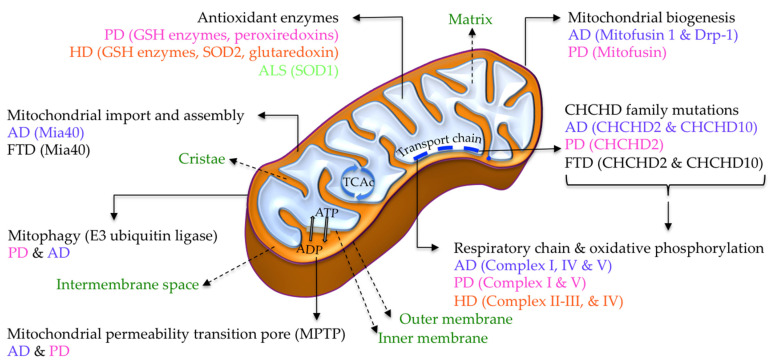
Mitochondrial SCCPs and cysteinet dysregulation in neurodegenerative disorders. Mitochondria are the primary origin of reactive species, which can redox modulate SCCPs into the organelle participating in its biogenesis, the mitochondrial permeability transition pore (MPTP), mitochondrial import and assembly (Mia) of proteins, and the bio-energetic ability involving enzymes of the tricarboxylic acid cycle (TCAc) and enzymatic complexes of the respiratory electron transport chain and oxidative phosphorylation. The initial insult may affect different SCCPs but would disturb mitochondrial homeostasis and the efficiency of ATP biogenesis. Alzheimer’s disease (AD), Parkinson’s disease (PD), Huntington’s disease (HD), frontotemporal dementia (FTD), and amyotrophic lateral sclerosis (ALS).

**Figure 3:**
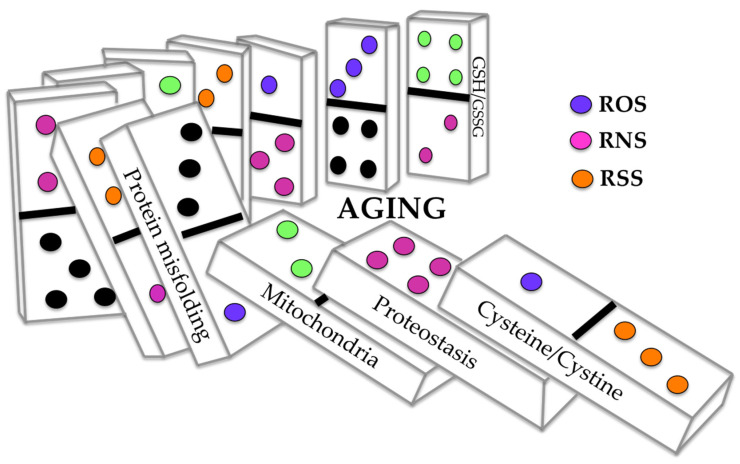
Cysteinet deregulation in aging and neurodegenerative disorders. Cysteinet is defined as a bottom-up cellular network integrated by reactive species, the cysteine/cystine and reduced/oxidized glutathione (GSH/GSSG) cycles, and all proteins containing functional cysteines. Sensitive-cysteine-containing proteins (SCCPs) participate in diverse metabolic, signaling, and structural processes but are modulated by the same thiol (-SH) radical. It is hypothesized that cysteine/cysteine ratio disturbance may initiate a domino effect leading in subsequent steps to deregulation of SCCPs including GSH/GSSG status. These SCCPs are redox altered (S-glutathionylation, S-nitrosylation, sulfenylation, disulfide bonds formation), producing reversible or irreversible changes in the protein physiological action, folding and accumulation when proper proteostasis is affected by the cysteinet disturbance. ROS (reactive oxygen species); RNS (reactive nitrogen species); RSS (reactive sulfur species); GSH (reduced glutathione); GSSG (oxidized glutathione). Black and green dots represent oxidized (-S-S-) and reduced (-SH) thiol groups associated with reactive cysteine residues in peptides and proteins.

The following sections review the critical role of the disturbance of sensitive-cysteine thiol groups in SCCPs from different cellular pathways and their integration in the proposed cysteinet deregulation contributing to proteostasis impairment in neurodegeneration (Figure 1, Figure 2 and Figure 3).

## 4. The Sensitive-Cysteine Redox Proteome (Cysteinet) in Neurodegenerative Diseases

Age-associated disorders, including neurodegenerative diseases, are accompanied by deregulation in redox protein homeostasis, mitochondrial dysfunction, macromolecular oxidative damage, and accumulation of misfolded proteins into different cellular compartments [1,6,7]. Site-specific thiol groups in reactive cysteine residues in proteins serve as redox sensors, and this sensitivity makes them work as switches to synchronize diverse biochemical pathway functions. This interactive type of post-translational modification by small molecules (ROS, RNS, and RSS) leads to the close regulation of thousands of proteins in the cytosol and specific organelle compartments in cells [1,6,7,11,12,13]. Redox-regulated proteins can suffer different types of reversible (palmitoylation, sulfenic acid) or irreversible (prenylation, sulfinic, and sulfonic acid) redox modifications in response to various small oxidants and compartmentalized subcellular pH [6,7,44]. Indeed, the cellular antioxidant machinery (thioredoxins, glutaredoxins, peroxiredoxins) uses reversible changes in the redox state of its catalytic cysteine residues to restore the redox status of different cellular proteins [44,45]. Therefore, cysteine redox-modifications of proteins are exquisitely regulated, participating in proper protein folding, function, and secretion through palmitoylation, prenylation, S-nitrosylation, and S-glutathionylation [44,45].

It has been demonstrated that the expression of amyloid fibrils induces a shift toward cytosolic oxidizing conditions in neuronal cells and vice versa, suggesting the existence of a complex signaling mechanism for redox state regulation [46], which we have proposed to be organized into a broad cellular and intercellular sensitive-cysteine network called cysteinet [1]. It remains to be clarified how cysteinet is regulated and deregulated in pathological conditions, including aging and neurodegenerative diseases. Small oxidizing and reducing molecules, such as hydrogen peroxide, nitric oxide, hydrogen sulfide, NADP/NADPH, free cysteine/cystine, and GSH/GSSG mediate this organism-broad redox network. The impairment of proteostasis in some cells could affect neighboring cells’ protein folding conditions, leading to redox homeostasis imbalances affecting protein transport between cells, disturbing the redox response in distinct compartments and tissues [1,46].

## 5. Alzheimer’s Disease

AD is the most frequent age-associated neurodegenerative disorder characterized by aberrant processing of the amyloid precursor protein (APP), increasing Aβ1-42 peptide accumulation, and formation of intraneuronal neurofibrillary tangles by the hyperphosphorylation of the microtubule-associated protein tau (MAPT) in the brain [47,48,49,50,51]. The accumulation of both proteins has been associated with neuronal loss and cognitive impairment in AD patients [52,53]. To integrate proteostasis impairment in AD, we have proposed that genetic, mitochondrial, toxic, metabolic, inflammatory, and age-related factors can converge in the deregulation of cysteinet that finally results in neuronal degeneration. Therefore, redox protection of SCCPs may contribute to stabilizing proteins against irreversible oxidative damage or revert them to their physiological state [54]. The reversibility of thiol changes permits cysteine residues in proteins to function as regulators in numerous regulatory routes and various structural sites in the cell. Nonetheless, the irreversibility of thiol oxidation causes cumulative deterioration of proteins with harmful effects [55,56].

Cysteinet works as a bottom-up biochemical network, formed by reactive species serving as modulators, the cysteine/cystine and GSH/GSSG cycles, and all peptides and proteins with functional cysteine residues in their structure [1,2,3]. Cysteine residues in SCCPs may act as cellular sensors engaging, at very short timescale, very different cellular functions [1,2,3,4,5].

### 5.1. Cysteinet Deregulation in AD

Diverse etiologic events can contribute to deregulating the age-associated cellular redox balance developing into cysteinet disturbance in age-related neurodegenerative diseases (Figure 3). ROS overproduction has been related to age-associated mitochondrial DNA injury and impairment in bioenergetic capability in patients that are homozygous for apolipoprotein E4, resulting in a dysfunction of cysteinet that can hamper diverse pathways, promoting, among others, Aβ amyloid and tau accumulation (Figure 1, Figure 2 and Figure 3).

#### 5.1.1. Apolipoprotein E (ApoE)

ApoE isoforms show arginine to cysteine differences in their N-terminal domains. ApoE4 contains two arginine residues at positions 112 and 158. However, ApoE3 contains a cysteine residue at position 112, while ApoE2 contains two cysteine residues at positions 112 and 158, respectively [57,58]. These amino acid substitutions in E apolipoproteins result in 3D structural changes with functional consequences [58]. Sensitive-cysteine residues in ApoE2 and ApoE3 may bind and detoxify the free radical 4-hydroxynonenal, a cytotoxic lipid peroxidation by-product. However, ApoE4 homozygosity may contribute to brain aging by its inherent decreased neuroprotective ability because of the absence of reactive cysteine residues in ApoE4 [59]. Therefore, it seems that ApoE isoforms have different redox capacities through sensitive-cysteine residues. ApoE2 and Apo E3 can bind to neuronal nitric oxide synthase (NOS1), and they can be S-nitrosylated in the human hippocampus, potentially regulating lipid metabolism in AD [60]. 

The apolipoprotein genotype E4 is the most notable genetic linkage to late-onset AD [61], and people with the E4 allele have a decreased lifespan [62] and increased chance of suffering from AD [63]. Aβ clearance drops from ApoE2 to ApoE3 to ApoE4, suggesting a connection between the clearance of Aβ and ApoE isoforms [64]. Cys112 in ApoE3 allows the formation of disulfide bridges between ApoE3 molecules forming monomers and homodimers [65], whereas Cys112 and Cys158 in ApoE2 enable the formation of monomers and homodimers, as well as homopolymers [65] (Figure 1). However, the absence of cysteine residues in ApoE4 does not permit the formation of disulfide bonds between ApoE4 molecules and potentially contributes to its decreased efficiency at clearing Aβ. The resulting Aβ accumulation can increase oxidative damage facilitating the onset of AD in APOE4 genotypes. Additionally, the high-temperature requirement protein A1 (HtrA1) is involved in the degradation of APOE4, Aβ, and hyperphosphorylated tau through redox-regulated mechanisms that involve sensitive-cysteine residues in the N-terminal third of the HtrA1 protein, resulting in a 3D structural modification that decreases its binding to APOE4, and possibly to Aβ and tau proteins [66].

#### 5.1.2. Mitochondrial SCCPs

AD brains show increased levels of reactive species affecting critical proteins for neuronal survival and contributing to disease pathophysiology [67]. Mitochondrial dysfunctions occur in AD brains before clinical symptoms and Aβ plaque formation [68,69,70,71,72]. It seems that synaptic mitochondria are specifically vulnerable in aging and AD [72,73,74], being the primary source of reactive species. Reactive species can significantly modulate SCCPs in the organelle participating in the bio-energetic ability of the cell, including the tricarboxylic acid cycle (TCAc), complexes of the respiratory electron transport chain, and oxidative phosphorylation (Figure 1 and Figure 2).

Aconitase and pyruvate dehydrogenase are mitochondrial enzymes redox-regulated by hydrogen peroxide and S-glutathionylation by the oxidation and reduction of specific sensitive-cysteine residues [54,75]. L-carnitine/acyl-carnitine carriers can be S-glutathionylated on Cys136 and Cys155 [75]. In addition, the enzyme succinate dehydrogenase can be S-glutathionylated in oxidizing conditions [75]. Redox modifications by S-glutathionylation are reversible and occur under stress-inducing and physiological conditions. During aging and other stress-inducing conditions with decreased GSH/GSSG balance in the mitochondria, the oxidative shift of these SCCPs may be irreversible.

Mitochondrial complex I of the respiratory chain can suffer reversible sulfenylation, decreasing its enzymatic activity (Figure 1 and Figure 2). Complex I sulfenylation may suffer further irreversible oxidative modification resulting in its inactivation [75]. However, sensitive-cysteine residues in complex I can be saved from additional oxidation by S-glutathionylation [54]. Therefore, the S-glutathionylation of complex I restricts NADH production, lowering the electron flow through the respiratory chain, decreasing ROS over-production, and protecting this enzymatic complex from irreversible oxidation. 

Cytochrome c oxidase (complex IV) is a crucial enzyme in the mitochondrial respiratory electron transport chain, containing fundamental cysteine residues that play basic roles in metal coordination required for the redox-linked proton pumping by the enzyme [76,77]. Mitochondrial complex IV activity is reduced in brain tissue, fibroblasts, and blood platelets from AD patients [76,77,78,79]. Additionally, oxidative phosphorylation is modulated through the redox change of the ATP synthase (complex V) by S-glutathionylation on Cys294 of the α-subunit placed in the F1 hydrophilic portion of the protein. In addition, Cys294 may form a disulfide bond with the neighboring Cys103 residue [80]. S-glutathionylation blocks nucleotide-binding to the complex resulting in a decrease in ATP generation. Thus, the oxidative modification of proteins in mitochondria can control ROS production and the bioenergetic ability of cells (Figure 1 and Figure 2).

Accumulation of β-amyloid inside brain mitochondria of AD patients interferes with mitochondrial fusion and fission, contributing to AD pathophysiology [81,82]. Since neuronal bio-energetic demand relies on mitochondrial dynamics mediated by fission and fusion processes to generate new mitochondria, the redox interference with these pathways is crucial. Among SCCPs that participate in mitochondrial biogenesis, the integral membrane GTPases mitofusin 1 and dynamin-related protein-1 (Drp-1) are two well-known redox-regulated proteins (Figure 1 and Figure 2). Indeed, the fragmentation of mitochondria and the resulting loss of synapses in AD patients have been associated with S-nitrosylated Drp1 increased levels [83,84,85]. Redox micro-environmental conditions also regulate mitofusin’s S-glutathionylation, which is required to induce mitochondrial hyperfusion [86,87], showing that mitochondrial SCCPs control the structure and biogenesis of mitochondria depending on the redox modulation of thiol groups in sensitive-cysteine residues of the involved proteins.

ROS overproduction can even alter the mitochondrial permeability transition pore (MPTP) through sulfenylation and S-glutathionylation modulating MPTP opening (Figure 1 and Figure 2), which requires the formation of disulfide bridges between distinct cysteine residues in the adenine nucleotide translocator (ANT), a member of MPTP that exports ATP from the mitochondrial matrix and imports ADP into the matrix [88,89,90].

#### 5.1.3. Cytosolic SCCPs

Many cytoplasmic proteins are modulated by cysteine redox modifications, including enzymes involved in glucose metabolism (Figure 1). Glyceraldehyde-3-phosphate dehydrogenase, pyruvate kinase, phosphofructokinase, glucose 6-phosphate isomerase, glycogen phosphorylase, phosphoglycerate mutase 1, and phosphoglucomutase 2 need cysteine residues for their action [91].

Glyceraldehyde-3-phosphate dehydrogenase (GAPDH) has been shown to be involved in AD pathophysiology [92]. Cytoplasmic GAPDH exists as a tetramer composed of four identical monomers containing a single sensitive-cysteine residue critical to the enzyme’s catalytic function [93,94]. This enzyme catalyzes the reversible phosphorylation of glyceraldehyde-3-phosphate involving the thiol group of Cys152 [92]. The four sensitive-cysteine residues of GAPDH can be oxidized by hydrogen peroxide, reducing the stability of the protein resulting in monomers, dimers, and other denatured products [92]. Similarly, under pathological conditions, GSH can react with Cys152 contributing to the formation of disulfide bridges [92]. GAPDH has been found oxidized in AD brains [92], though various investigations have indicated that S-glutathionylation of the enzyme is a mechanism to protect the protein against irreversible damage in the oxidizing environment of the AD brain [92]. Likewise, S-nitrosylation of Cys152 in GAPDH can be reversibly modified, inhibiting its dehydrogenase activity [92,93,94]. Interestingly, Cys152 in the enzyme’s active site is required to induce apoptosis by oxidative stress [94].

#### 5.1.4. Protein Tyrosine Kinases (PTK)

Protein tyrosine kinases (PTK) are a superfamily of enzymes containing conserved sensitive-cysteine residues (Figure 1). For instance, protein tyrosine phosphatase 1B is redox-regulated by cysteine residues located in the catalytic core of the enzyme that are reversibly oxidized by hydrogen peroxide and by S-glutathionylation [95]. The result of these oxidative changes is an increase in tyrosine phosphorylation [95]. A subset of the PTK superfamily, including three of the ten Src family kinases and all four kinases of the FGFR family, have a sensitive-cysteine residue (Cys277) located in the catalytic domain. These PTK are vital enzymes in mammalian signal transduction pathways, and the reversible oxidative modification of Cys277 directly regulates PTK in the Src and FGFR families [96]. The aberrant protein phosphorylation in AD involves numerous examples of altered protein phosphorylation pathways, including protein tyrosine kinase (PTK). Several FGFs and their receptors (FGFR) are involved in the pathogenesis of AD [97,98,99,100,101]. Over-expression of FGF2 in AD patients repairs spatial learning, long-term potentiation, and neurogenesis, likely mediated by FGFR1-activated boosts in the OX-2 membrane glycoprotein (CD200). This OX-2 membrane glycoprotein regulates microglial activity and promotes neurite outgrowth and neuronal survival, suggesting a pivotal role of FGFRs in the crosstalk between degenerating neurons, microglia, and astrocytes [101].

#### 5.1.5. Synaptic SCCPs

Reduced expression of the cysteine string protein α (CSPα) in forebrain areas in post-mortem samples from AD patients has been reported [102]. CSPα is a protein that regulates vesicle endocytosis participating in synaptic transmission and the maintenance of synapses [103]. The oligomerization of CSPα depends on a cysteine-rich “string” region of the protein for its attachment to synaptic vesicles [103].

The frontal cortex in AD patients show significantly decreased synaptophysin concentrations than controls [104]. Synaptophysin plays a central role in the synaptic alterations found in AD, and its loss in the hippocampus correlates with cognitive decline in AD patients [105]. The role of this protein in the synaptic vesicle membranes is the formation of channels that depend on redox modifications in specific sensitive-cysteine residues. These sensitive-cysteine residues form disulfide bridges that connect with neighboring synaptophysin monomers (Figure 1). Synaptophysin cross-linking by disulfide bonds is modulated by an exquisite redox regulation, suggesting that native synaptophysin depends on the fine building of cysteine-associated disulfide bonds to create multimeric complexes in the precise phospholipid environment [106].

More than 50% of AD patients show α-synuclein cumulation [107]. Though human α-synuclein does not include any cysteine residues [108], tyrosine to cysteine substitution at vital places in the α-synuclein protein can raise dimer formation, accelerating protein aggregation and cellular toxicity of α-synuclein [109]. α-synuclein may also function as a chaperone-like protein in synergy with CSPα for assembling the SNARE (soluble NSF attachment protein receptor) complex [110]. This α-synuclein function involves linkage with synaptotagmin proteins, which have sensitive-cysteine residues in their configuration [110,111].

#### 5.1.6. APP Processing, Aβ Aggregation

Familial AD is associated with APP, presenilin-1, and presenilin-2 gene mutations [112]. The demonstration that transgenic mice expressing familial human APP and presenilin mutations present significant features of the human AD [113] supports the amyloid cascade hypothesis [114], suggesting that the abnormal processing of APP leads to the accumulation of Aβ peptides. Besides, the hyperphosphorylation of tau appears to be a crucial element for AD outcome [113,114].

Human presenilin-1 includes five cysteine residues [115], three in transmembrane domains, and two uncovered to the cytosol. Cys410 and Cys419 in presenilin-1 transmembrane domain 8 can connect with Cys92 in transmembrane domain 1, contributing to the protein’s conformational modifications enabling their reaction with the active site of γ-secretase [116]. These active cysteine residues may sustain the integrity and hydrophilic background necessary for the active center functionality [116]. Interestingly, familial AD patients have exhibited mutations in three of the five cysteine residues in presenilin-1, indicating the relevance of sensitive-cysteine redox deregulation in AD pathophysiology in early-onset cases [117,118] (Figure 1). In contrast to presenilin-1, disulfide bonds between Cys14-Cys31 and Cys56-Cys65 in presenilin-2 are critical determinants of the 3D structure of the protein. These sensitive-cysteine residues are also crucial in regulating ryanodine receptor (RYR)-mediated Ca^2+^ release [119].

Sensitive-cysteine residues may participate in the palmitoylation of proteins, forming a thioester bond between the cysteine and palmitic acid, raising the hydrophobicity of the protein and promoting its integration into cellular membranes [120]. The withdrawal of APP from the ER requires two palmitoylation sites at Cys186 and Cys187 placed within the copper-binding part of APP, stabilizing the domain configuration and constructing disulfide bridges with Cys158 and Cys133, respectively [121] (Figure 1). Accordingly, increased APP palmitoylation enhances the non-amyloidogenic β-cleavage of APP [121].

#### 5.1.7. Microtubule Associated Protein Tau (MAPT)

The imbalance between protein kinases and phosphatases leads to tau’s hyperphosphorylation. The pathological aggregation of tau-forming paired helical filaments (PHFs) and neurofibrillary tangles also depends on the intermolecular cross-linking of Cys322 [122]. Different reactive species can cause cysteine oxidation in tau and microtubule-associated protein-2 (MAP2), changing the capacity of these proteins to interact with microtubules [123] (Figure 1). Thus, augmented oxidative modification of sensitive-cysteine residues encountered in brain aging may contribute to tau and other protein aggregation in AD patients [122].

Tau shows auto-acetylation activity through a couple of catalytic active cysteine residues in its microtubule-binding domain [124] (Figure 1). Tau proteins are nearly 99% attached to microtubules in adult neurons [124], inhibiting their tau acetyl-transferase activity, blocking the functional cysteine residues connected to microtubules. Thus, microtubule detachment and the consequent activation of tau auto-acetyl transferase activity illustrate a pathological event which increases the pool of aggregation-prone tau species [124]. Consistent with this idea, acetyl-CoA levels are reported to be elevated in AD brains [124], supporting the role of increased activity of tau acetyltransferase in the disease’s progression. Indeed, tau aggregation inhibitors, such as methylthioninium, prevent tau filament formation via oxidation of cysteine residues in the tau repeat domain, preventing formation of disulfide bonds and maintaining the tau protein in a monomeric conformation [125]. Further, molecules that attach reactive cysteine residues of tau can stop neurofibrillary tangle-associated brain dysfunction [126].

#### 5.1.8. SCCPs in Calcium Homeostasis

AD patients also show calcium (Ca^2+^) disturbances attributed to Aβ-amyloid and NMDAR channel upregulation and mechanisms that implicate the prion protein [127,128]. Intra-neuronal Ca^2+^ levels depend on the balance between cytosolic Ca^2+^ pumping into the ER lumen by the ER Ca^2+^ ATPase (SERCA) pump and the discharge of Ca^2+^ from the ER through the inositol 1,4,5-trisphosphate receptor (IP3R) and the RYR. RYR amplifies the IP3R-mediated clearance of Ca^2+^ from ER, improving neuronal Ca^2+^ signals [129]. IP3R and RYR receptors are the principal neuronal Ca^2+^ channels regulated by the redox modification of sensitive-cysteine residues (Figure 1). S-nitrosylation and S-glutathionylation of various sensitive-cysteines control the closed state of the RYR1 complex resulting in a shift in Ca^2+^ release to the cytoplasm [130]. In addition, IP3R contains numerous functional cysteine residues modified by ROS delivered in the ER and the mitochondria [131]. This receptor controls cytoplasmic Ca^2+^ concentrations by modulating specific sensitive-cysteine residues, adjusting the active 3D conformation of the receptor [132].

Previous results are relevant because IP3R levels are reduced in the hippocampus of AD patients, indicating substantial correlations with the senile plaque stage and neurofibrillary tangle pathology [133]. Additionally, Cys674 in SERCA can be oxidized by high glucose concentrations, preventing Cys674 inhibition by nitric oxide and hydrogen peroxide [134], consequently contributing to Ca^2+^ disturbances in AD (Figure 1). Experiments with the skeletal muscle of hybrid rats showed that the SERCA pump is specifically oxidized in sensitive-cysteine residues during aging, suggesting age-related decrease in SERCA pump activity and, therefore, age-associated decrease in Ca^2+^-ATPase activity [135].

#### 5.1.9. SCCPs and Misfolding

The 3D structures of proteins are closely related to physiological and pathological consequences. Aging and other stressful conditions can disregulate the exquisite equilibrium among protein synthesis, folding, and degradation, contributing to protein aggregation. Protein folding depends on the primary sequence and cysteine protein content to form physiologically structural disulfide bridges under distinct redox situations [136]. Cysteine thiol groups of proteins can change between oxidized and reduced forms, contributing to their folding and unfolding configuration [137]. Many misfolded proteins contain disulfide bonds that can be irreversible [138]. In the case of SCCPs, the spontaneous folding process can be prolonged, requiring the formation of disulfide bonds [139]. Consequently, in vivo disulfide bridge-building is catalyzed by specific enzymes, such as PDI (Figure 1) [140]. However, the normal PDI enzyme was found S-nitrosylated in brain specimens from patients with PD and AD [141], facilitating further oxidative modifications of many other proteins and their accumulation in the ER, and compromising the chaperone function in protein folding [141].

As previously mentioned, mitochondrial biogenesis is dependent on protein import from the cytoplasm by mitochondrial translocation routes. The mitochondrial import and assembly (MIA) pathway guides proteins to the intermembrane space (IMS), coupling the transport to folding and oxidation mechanisms that result in the covalent modification of the incoming protein predecessor incorporating disulfide bridges in the procedure [142]. Indeed, Mia40 (a thiol oxidase not belonging to the thioredoxin family) has its catalytic disulfide site arranged in a unique Cys-Pro-Cys motif, allowing the initially mixed disulfide formation between Mia40 and the substrate protein to drive the oxidative folding process that depends on the specific sensitive-cysteine residues to form the initial enzyme-substrate-mixed disulfide [143]. Therefore, Mia40 and other redox-active proteins, such as thioredoxin, glutaredoxin, and peroxiredoxin, participate in the redox-dependent chaperone-like mechanisms in the mitochondrial IMS, which are associated with specific sensitive-cysteine residues [143] (Figure 1 and Figure 2).

#### 5.1.10. SCCPs in the Ubiquitin-Proteasome

The ubiquitin-proteasome machinery identifies and degrades impaired proteins, including misfolded and aggregated proteins [144]. The ubiquitin-proteasome machinery is a multistep pathway to recycling cellular proteins involving ATP-dependent activation of the ubiquitin protein that utilizes two enzymes (E1 and E2) to form ubiquitin-E2, binding to the target protein through the intermediation of the ubiquitin-protein ligase (E3) [145]. These phases depend on sensitive-cysteine residues that assemble successive thioester bonds concluding with the protein degradation in the proteasome [145]. Hence, the ubiquitin-proteasome system depends on the redox modifications of the thiol groups of functional cysteine residues of the proteins involved in the pathway [146,147] (Figure 1). Accumulation of ubiquitinated protein aggregates occurs during physiological brain aging, reaching pathological grades in various neurodegenerative disorders, such as AD [148,149,150,151]. Similarly, a mouse model with spatial memory deficits and decreased brain proteasome activity showed increased levels of various proteins typically accumulated in AD brains [152].

#### 5.1.11. Cysteine Proteases

Cysteine proteases, including calpains, cathepsins, and caspases, can play important roles in AD pathophysiology [153]. Calpains are cysteine proteases involved in AD pathophysiology via reactivation mechanisms, including enriched intracellular Ca^2+^ levels [154]. Moreover, Aβ peptides can activate calpains by increasing Ca^2+^ concentrations contributing to neuronal cell disruption and death [125]. Calpain 1 and 2 activation depends on Ca^2+^, allowing the rearrangement of cysteine, histidine, and asparagine in the catalytic center [155] (Figure 1). Thus, Ca^2+^ binding and the oxidation form of functional cysteines in the catalytic center are necessary for these proteases adequate activities [156].

It has been shown that many proteins of the caspase family are transcriptionally elevated in AD, playing a role in AD pathophysiology [153]. Additionally, the S-nitrosylation of functional cysteines on the catalytic hub of caspase-3 can prevent apoptosis in AD [157]. Similarly, inhibitors of apoptosis (IAPs) are a family of proteins that control cell survival through the attachment to caspases inhibiting their catalytic actions [158]. The X-linked inhibitor of apoptosis (XIAP) protein is the principal cellular caspase inhibitor among the IAPs, and it is S-nitrosylated in various neurodegenerative diseases. Accordingly, exciting studies have shown significant increases in S-nitrosylated XIAP in brain specimens from PD, AD, and HD patients, potentially boosting apoptosis [159] (Figure 1).

#### 5.1.12. Transcription Factors as SCCPs

Redox regulation of cysteine residues participates in the regulatory machinery of some gene transcription. For example, disulfide binds govern the building of the homotrimer of the heat shock transcription factor 1 (HSF1) that is translocated to the nucleus, activating the transcription of the heat shock proteins (Hsp) 70 and Hsp90. Hsp70 and Hsp90 have been shown to be involved in AD pathophysiology [160]. Under oxidative conditions, the molecular disulfide bridge prevents the action of HSF1, whereas under reducing conditions, these molecular links are split, and HSF1 cannot assemble the necessary trimer [160] (Figure 1).

AD brains show a decrease in the expression of the transcription factor Nrf2 and related pathways, interfering with diverse pathogenic processes, including Aβ and tau pathway deregulation [161]. Nrf2 modulates the expression of antioxidant proteins defending against oxidative injury and regulating the inflammatory response. Nrf2 is maintained in the cytoplasm by the Keap1 and Cullin-3 proteins, allowing the degradation of Nrf2 by ubiquitination [162]. Therefore, oxidative modification of vital sensitive-cysteines in Keap1 disrupts the Keap1-Cullin-3 transcription factor in the ubiquitination pathway, blocking Nrf2 ubiquitination. Thus, Nrf2 translocation into the nucleus allows interaction with the musculoaponeurotic fibrosarcoma (Maf) protein, activating the antioxidant response of many antioxidant genes and initiating their transcription [163].

## 6. Parkinson’s Disease

PD is an age-associated degenerative disorder distinguished by dopaminergic neurodegeneration in the substantia nigra of the brain [164], also affecting the locus coeruleus that shows intraneuronal Lewy bodies composed of aggregates of α-synuclein and other proteins [165] intermixed with intracellular membranous organelles [166]. The localization of Lewy bodies and neuronal dopaminergic loss in PD suggested that α-synuclein aggregation and deposition are responsible for the disease. Nonetheless, Lewy bodies occur in the brains of asymptomatic, aged individuals [167]. Recent data shows that the accumulation of α-synuclein starts as micro-aggregates of the protein at the presynapse, impairing neurotransmitter vesicle trafficking and discharge in dopaminergic neurons [168,169]. It has been proposed that Lewy bodies are formed to rescue neurons from the harmful outcomes of protein misfolding [170].

Analogously to AD, only 5% of PD patients show mutations in a few genes, but most cases are sporadic [171,172,173]. The increasing proteomic intricacy of sporadic PD has been demonstrated in the last ten years, suggesting that very distinct biochemical pathways underlie this disorder’s clinical manifestation and progression [5,171,172,173,174]. Oxidative impairment, reduced antioxidant ability, and mitochondrial dysfunction in the dopaminergic system have been shown to be involved in PD pathophysiology [175,176,177].

Sporadic PD patients are associated with exposure to harmful compounds, including pesticides, such as paraquat, that impair mitochondrial complex I activity, inducing increased ROS production in neurons. Therefore, oxidative damage and individual mitochondrial genetic susceptibility contribute to PD pathophysiology, modifying the functionality of various proteins, including those of the bioenergetic metabolism [178,179,180]. Since ROS are products of dopamine metabolism in dopaminergic neurons modulating intermediary and bioenergetic metabolism [181], cellular antioxidant machinery is indispensable for preserving redox homeostasis. Reduced concentrations of GSH appear to be the initial biochemical event recognized in the substantia nigra of premature PD individuals, correlating with PD severity and contributing to mitochondrial protein damage by oxidative insults [179]. Decreased GSH is the earliest indicator of associated mitochondrial complex I deficiency in dopaminergic neurons [178,180]. Senescence can even participate in this process by accumulating oxidative injury, reducing antioxidant capacity, and decreasing mitochondrial bioenergetic capacity in the brain by protein oxidation mechanisms [182,183,184,185,186,187].

### 6.1. Cysteinet Deregulation in PD

Recent investigations, including prioritized protein-protein relation networks, have revealed that proteins implicated in PD pathophysiology are involved mainly in the autophagy-lysosome and α-synuclein aggregation pathways [188]. These results reinforce the notion that the proteome in PD involves proteostasis failure by mechanisms that remain unknown. Global quantification of reduced and oxidized cysteine residues in physiological conditions has shown that, in the substantia nigra, the dopaminergic neurons are more oxidized than in the ventral tegmental region [189,190]. Accordingly, some investigations support the view that oxidized cysteine residues in dopaminergic neurons of the substantia nigra in PD are more oxidized than in other areas [191,192,193]. These results suggest that the disturbance of the sensitive-cysteine proteome in PD plays a crucial function in its pathophysiology and advancement [1,2,3,4,5,194] (Figure 1, Figure 2 and Figure 3).

As previously mentioned, proteostasis involves vital pathways that support protein homeostasis, including secretion via extracellular vesicles transporting diverse proteins among cells. Most cells can secrete small vesicles (exosomes and microvesicles) through various energy-dependent enzymes, which transport a complex mixture of proteins, nucleotides, and lipids [195]. Some analyses have shown that cells under oxidative stress sustain redox homeostasis by discharging cysteine-oxidized proteins through exosomes [195,196], such as the thiol-dependent peroxiredoxins [197]. The cysteine oxidized enzyme GAPDH can be released by exosomes [195], implying membrane-bound and intraluminal protein mechanisms recognizing oxidized-cysteine residues in those proteins released by exosomes as a protective mechanism against oxidative damage. Similarly, microvesicle and exosome discharge rely on cytoplasmic Ca^2+^ inflow through various Ca^2+^ channels that contain functional cysteines triggered by oxidation [198]. For instance, exosome discharges under oxidative states depend on the particular thiol oxidation of IP3R, triggering calcium release from the ER to the cytoplasm [195]. Furthermore, Ca^2+^-dependent stimulation of calpains enables microvesicle building under the redox modification of sensitive-cysteines [199] (Figure 1, Figure 2 and Figure 3).

#### 6.1.1. DnaJ Homolog C (DNAJC) Family

The propagation of α-synuclein among neurons occurs through interactions with protein partners [200,201] and via exosomes [202]. Inside the new neuron, α-synuclein fibrils are transported via axons to the nearest neurons or removed by the ubiquitin-proteasome system [203,204]. DNAJC proteins are a subclass of the heat shock protein (HSP) family, mutations in which are associated with PD and other neurodegenerative diseases displaying Parkinsonism [205,206]. CSPα is encoded by DNAJC5 and is abundantly localized in presynaptic vesicles [205,206]. α-synuclein cooperates with CSPα to maintain the soluble SNARE complex assembly, which fuses vesicles with the target membrane [207,208,209]. CSPα includes a string-domain cysteine containing 13–15 heavily palmitoylated cysteines (Figure 1). These palmitoylated cysteines are required for fusion goals, and modifications in this domain result in high molecular weight aggregates of CSPα, indicating that a set of palmitoylated cysteines is necessary for the assemblage of CSPα [210].

Additionally, it has been shown that increased neuronal activity increases the release of tau through exosomes, promoting its propagation to neighboring neurons [211,212]. Similarly, the spreading of tau by exosomes is raised in PD, implying that the dissemination of some proteins among neurons and between neurons and glial cells may play a role in progression of the illness [213].

#### 6.1.2. Glucocerebrosidase (GCase) 

About 10% of patients with PD carry a mutation of the lysosomal enzyme glucocerebrosidase (GCase), which has a crucial role in Gaucher disease. However, only some carriers with GCase mutations develop PD [214,215]. Moreover, some cases of idiopathic PD, without GCase mutations, show reduced enzyme concentrations [214,215], suggesting potential interactions between autophagy disturbances and α-synuclein accumulation in PD. Physiological wild-type GCase concentrations may ameliorate the phenotypes of Parkinson’s disease models. GCase activation in pluripotent-stem-cell-derived dopaminergic neurons from sporadic PD subjects enhances the lysosomal role and diminishes the pathological aggregation of oxidized dopamine, glucosylceramide, and α-synuclein [216]. Analogous effects have been observed in patients harboring mutations in the genes encoding the GCase, LRRK2, DJ-1 (PARK7), or parkin, showing reduced GCase functionality [216]. Since mutations in GCase are a considerable genetic risk characteristic for PD, it was proposed that the activation of wild-type GCase is a probable restoring therapy for those cases of PD exhibiting impaired GCase activity, including familial instances of PD related to mutations in other cited proteins [216].

GCase contains two disulfide bonds and three free thiolic groups in cysteine residues necessaries for the GCase activity, which serve as molecular modulators by glutathione [217] (Figure 1). Moreover, increased oxidized dopamine can regulate GCase acting at sensitive-cysteines within its functional domain [218], suggesting potential oxidative modification of sensitive-cysteine residues within GCase [217,218]. Mass spectrometry analysis revealed 28 sensitive-cysteine residues, 23 in different GCase domains. Only Cys1465 is in the GTPase domain, and four (Cys2024, Cys2025, Cys2101, and Cys2114) are in the kinase domain of the protein [219].

#### 6.1.3. Leucine-Rich Repeat Kinase 2 (LRRK2)

The LRRK2 gene encodes the kinase enzyme leucine-rich repeat kinase 2 (LRRK2), also known as PARK8. The enzyme participates in cell autophagy and is present in the cytoplasm and the mitochondrial outer membrane. LRRK2 contains two essential cysteine residues for redox sensing [220] in the activation loop of the protein (Figure 1). Variants of this gene participate in PD evolution, and over one hundred additional mutations in this gene can raise the probability of PD [221]. In idiopathic PD patients, ROS can activate LRRK2 in dopaminergic neurons, suggesting that oxidative change of the LRRK2 protein potentially produces the phosphorylation of its Rab10 substrate, resulting in a sequence of events ending in neuronal death [222]. Therefore, LRRK2 mutations in familial PD cases increase activation of the protein and participate in the impairment of sporadic PD, indicating that the dysregulation of the activity of this protein is implicated in PD pathophysiology [222]. 

Sporadic PD and LRRK2-related PD patients show decreased concentrations of 2-hydroxybutyrate in CSF compared to controls [223]. This metabolite is derived from threonine or methionine metabolism via homocysteine and cystathionine, generating cysteine that reestablishes GSH levels and glutathione synthesis [223].

#### 6.1.4. Cellular-Abelson Tyrosine Kinase (c-Abl)

The c-Abl protein plays a fundamental role in the cellular reaction against oxidative stress through sensing ROS [17,224]. The protein has key sensitive-cysteine residues that mediate the formation of mixed disulfide bridges inhibiting c-Abl kinase activity (Figure 1), indicating that the redox regulation of sensitive-cysteines in c-Abl is crucial for its kinase activity [225]. Recent studies show that c-Abl activation occurs in PD pathophysiology through the direct phosphorylation of α-synuclein, facilitating its accumulation and impeding its clearance by the ubiquitin-proteasome system [224]. Furthermore, parkin phosphorylation via c-Abl may inactivate the ligase activity of parkin, disrupting the proteasome function and enhancing dopaminergic neurodegeneration [224]. Consequently, c-Abl inhibitors have been proposed in the treatment of PD [224,226].

#### 6.1.5. Parkin

E3 ubiquitin ligase (Parkin) is a member of the RBR (RING-between-RING) ubiquitin ligase family that participates in ubiquitination mechanisms in proteasomes or lysosomes. RING0, RING1, RING2, and the in-between-RING (IBR) parkin subdomains are cysteine-rich and bind to eight Zn^2+^ ions [227]. Besides the catalytic Cys431, the redox reactive Cys268 and Cys323 identify impaired proteins on the outer mitochondria membrane mediating mitophagy [228,229]. Parkin’s sensitive-cysteines react with nitric oxide to generate S-nitrosylated parkin, which is increased in the brains of patients with Lewy body disease and PD because of interference with its neuroprotective function [230] (Figure 1). S-nitrosylation intervenes in parkin’s vulnerability to dopamine oxidative injury by modifying two functional cysteines on the protein (Cys268 and Cys323), different from other cysteine-bearing enzymes in the same family [230]. S-nitrosylation of parkin enhances neuronal survival blocking apoptosis [231,232]. This protein mutation is related to mitochondrial disturbances contributing to neuronal loss in PD [233]. Moreover, parkin can be transferred into the mitochondria under oxidative stress conditions without DJ-1 [227], a cellular controller of ROS that protects mitochondria [234].

#### 6.1.6. Dopamine Transporter (DAT)

DAT (dopamine transporter) is a transmembrane protein that is involved in the reuptake of the neurotransmitter dopamine at presynaptic terminals for further release. DAT contains eight sensitive-cysteines in the hydrophilic coils of the protein strategically positioned on both flanks of the cytoplasmic membrane. Specific cysteines are vital for preserving the adequate 3D conformation of the dopamine translocation transporter [235], while other cysteines act in the S-palmitoylation of the protein via thioester bridges [236] (Figure 1). Hence, DAT controls dopamine signaling and contributes to dopamine homeostasis [237].

#### 6.1.7. Protein Deglycase DJ-1 (Parkin7)

The PARK7 gene encodes the DJ-1 protein, a cysteine protease whose mutations produce autosomal-recessive PD patients and which also participates in sporadic PD cases [238]. Patients with sporadic PD and AD show inactive forms of DJ-1 by oxidative impairment. DJ-1 includes three functional cysteines (Cys46, Cys53, and Cys106) sensitive to S-nitrosylation, but Cys106 redox-modification mainly modulates protein activity (Figure 1). Thus, S-nitrosylation of DJ-1 disturbs its antioxidant function in dopaminergic cells [239]. Additionally, Cys106 S-nitrosylation in DJ-1 inhibits transnitrosylation to PTEN, increasing its phosphatase action and reducing neuronal survival [240].

#### 6.1.8. ATP-Sensitive Potassium Channel (K-ATP Channel)

K-ATP channels are complex proteins comprised of four potassium channels forming the pore and four sulfonylurea receptors (SUR1 or SUR2). Hydrogen sulfide (H_2_S) drives dopamine discharge through the redox change of two functional cysteines (Cys6 and Cys26) located in the N-terminal part of the regulatory subunit SUR1 of the protein complex [241,242] (Figure 1). Since redox activation of K-ATP channels modulates dopamine excitability, these channels have been shown to be involved in particular susceptibility to dopamine neurodegeneration in PD models [243].

#### 6.1.9. Antioxidant Enzymatic System

In addition to the glutathione enzymatic system, sensitive-cysteine residues also regulate other critical antioxidant enzymes. Peroxiredoxins are a group of antioxidant enzymes with a reactive cysteine placed in the active zone that reduce cellular peroxides with the participation of the thioredoxin system (Figure 1 and Figure 2). Peroxiredoxin 2 (Prx2), the most significant member of the peroxiredoxin family in mammalian neurons, has been found to be more S-nitrosylated in the brains of PD than in controls [244]. Oxidative changes at Cys51 and Cys172 in Prx2 form an intermolecular disulfide bridge with another Prx2 protein, which can be reversed by the thioredoxin system. Nevertheless, additional oxidation of Cys51 and Cys172 can block Prx2 inhibition of the antioxidant function against hydrogen peroxide, contributing to dopaminergic neuronal death [244].

#### 6.1.10. Mitochondrial Respiratory Chain and Oxidative Phosphorylation

Diverse Krebs cycle enzymes can suffer redox modifications in cysteine thiol groups. Some irreversible thiol-oxidative modifications can be deleterious for specific respiratory chain complexes resulting in additional ROS overproduction [75]. ROS, RNS, and RSS can modify mitochondrial complexes I, II, III, IV, and complex V of oxidative phosphorylation. Since complex I is the access point for electrons in the mitochondrial respiratory chain, the irreversible impairment of complex I enzymatic activity disrupts the electron flux, resulting in increased ROS generation and decreased ATP production (Figure 1 and Figure 2). Cysteine disulfide bridge construction between mitochondrial complex V subunits can significantly reduce ATP synthesis. Indeed, the disulfide link between Cys294 and Cys103 may interfere with its activity (Figure 1 and Figure 2). The redox transformation of Cys294 in the α-subunit by S-glutathionylation or S-nitrosylation may disrupt nucleotide-binding activity, reducing ATP synthesis [75].

#### 6.1.11. Microtubule-Associated Protein Tau and MAP2

Specific functional cysteine residues can facilitate tau dimerization and oligomerization at the microtubule-binding domain of the protein involved in the tau-tau connection [126]. Cys322 can be redox modified, regulating tau self-assembly and permitting the pathological accumulation of tau into paired coiled filaments and neurofibrillary tangles that spread under oxidative situations [122,245] (Figure 1). Vitamin B12 can interact with tau protein, blocking the reactive cysteines and interfering with tau aggregation [246]. Therefore, besides the imbalance between protein kinases and phosphatases that leads to tau fibrillation in neurodegenerative diseases, age-related and other disease-specific factors can increase cysteine oxidation in neuronal cells, increasing tau fibrillation. Peroxynitrite and H_2_O_2_ may also cause sensitive-cysteine oxidation in tau and MAP2, disturbing their assembly with microtubules and permitting their pathological accumulation [123] (Figure 1).

Tau protein can also catalyze its auto-acetylation through reactive cysteines in its microtubule-binding region (Figure 1), increasing insoluble tau aggregation by intramolecular and intermolecular acetylation reactions [124]. Under physiological conditions, 99% of tau in mature neurons is attached to microtubules, inhibiting tau acetyl-transferase action through the blockage of sensitive-cysteines. Consequently, tau auto-acetyl transferase activity represents a pathological occurrence contributing to cytosolic tau aggregation [124]. Interestingly, methylthioninium can prevent the building of tau filaments and their harmful precursors via oxidative modification of the cysteine residues controlling the formation of disulfide bonds and retaining the tau protein in a monomeric conformation [125]. Accordingly, compounds that bind to tau sensitive-cysteine residues can prevent neurofibrillary tangle-associated brain disruption [126]. Therefore, the suggested cysteine-mediated impaired redox modulation of essential proteins implicated in PD may also impact the accumulation mechanisms that participate in the advancement of the disease.

#### 6.1.12. α-Synuclein and the Ubiquitin-Proteasome

α-synuclein mediates vital roles in neuronal synaptic terminals holding neurotransmitter generation and reuptake, vesicle storage and motility, and mitochondrial homeostasis. Therefore, misfolding and aggregation of α-synuclein result in mitochondrial and bio-energetic dysfunctions and raised oxidative deterioration in dopaminergic neurons. However, the exact consequences of α-synuclein misfolding, assemblage, and toxicity are unknown. ROS and RNS can cause α-synuclein aggregation by generating durable cross-linking dimers [247]. Because α-synuclein does not contain cysteine or tryptophan amino acids in its structure, oxidative changes must impact tyrosine and methionine residues, including diverse effects in protein fibrillation and folding [247]. Point mutations in α-synuclein may increase its assembly by changing the secondary structure of the protein that, in addition to ROS overproduction in the dopaminergic metabolism, may contribute to protein misfolding and aggregation [248].

Oxidized α-synuclein is not optimally cleared by the ubiquitin-proteasome, contributing to its aggregation in neurons when GSH is reduced [170,249]. The ubiquitin-proteasome mechanism itself is disturbed by sensitive-cysteine dysfunction in PD since this system utilizes the successive activity of diverse SCCPs. The E1 enzyme initiates an energy-rich thioester bind that concerns the C-terminal glycine of the ubiquitin protein and the sensitive-cysteine of the functional center of the E1 enzyme, allowing the proteasome to identify and downgrade the ubiquitin-labeled protein [250]. E3 ubiquitin ligase (parkin) is modulated too by sensitive-cysteines participating in managing mitochondrial proteins by the proteasome [228].

The proteasome also activates transcription regulators, such as the NF-κB signaling pathway. Specifically, oxidative stress activates the NF-kB pathway and suppresses autophagy and autophagy-dependent apoptosis, permitting the assembly and outspread of α-synuclein [251]. Recent results showed that α-synuclein accumulation in mitochondria is associated with defects in cellular respiration [252]. Moreover, α-synuclein interferes with mitochondrial fusion sensitive-cysteine proteins, such as mitofusin-1 (Mfn1), mitofusin-2 (Mfn2), and optic atrophy type 1 (Opa1), promoting mitochondrial fragmentation [253] (Figure 1, Figure 2 and Figure 3).

## 7. Amyotrophic Lateral Sclerosis

Amyotrophic lateral sclerosis (ALS) is a degenerative disease without effective therapy characterized by progressive motor neuron death of the motor cerebral cortex, brainstem, and spinal cord, associated with muscle atrophy and paralysis [254]. Like other neurodegenerative diseases, ALS can be sporadic (90%) or familial in patients with mutations in genes coding for proteins implicated in diverse cellular processes, including deficient RNA metabolism, glutamate excitotoxicity, disorders of membrane trafficking, endoplasmic reticulum (ER) stress, mitochondrial deficiencies, and protein misfolding and accumulation [255,256]. The existence of motor neuronal inclusions developed by misfolded aggregated proteins is related to synaptic loss and neurodegeneration [254,257]. Notably, subjects carry mutations in the genes encoding the antioxidant enzyme superoxide dismutase1 (SOD1), a zinc and copper metalloenzyme that dismutates superoxide radicals to hydrogen peroxide. Other proteins, such as the RNA-binding protein trans-active response DNA-binding protein (TDP43), and the fused in sarcoma/translocated in liposarcoma protein (FUS/TLS), have protein inclusions of SOD1, TDP43, and FUS. It is noteworthy that TDP43 is also accumulated in sporadic ALS and non-TDP43 familial ALS cases, excluding those with SOD1 mutations [258].

### 7.1. Cysteinet Deregulation in ALS

Previous papers have shown evidence supporting the idea that ALS also causes deregulation of the sensitive-cysteine redox proteome [259], suggesting that redox deregulation can modify the 3D structure of proteins leading to the formation of cysteine-mediated protein aggregation and inclusions into neurons [259] (Figure 1, Figure 2 and Figure 3).

#### 7.1.1. Superoxide Dismutase

SOD1 has various disulfide bonds and free cysteine residues that contribute to its 3D structure, interaction with other molecules, and activity [260]. There are multiple sites where SOD1 can be oxidized: sensitive-cysteine residues, Trp32, and the copper site histidines [260]. The human native SOD1 contains four sensitive-cysteines, Cys57 and Cys146, composing an intra-monomer disulfide bond, whereas Cys6 and Cys111 are free (Figure 1 and Figure 2). Structural disulfides play critical roles in stabilization and dimerization, as is the case for the Cys57-Cys146 disulfide, anchoring the zinc loop to the active center of the protein [261] and increasing its thermal stability [260,261]. Cys111 is a pivotal regulator of SOD1 expression, folding, stability, assembly, and toxicity [259,260], and is set on the protein exterior close to the dimer interface allowing the formation of covalent disulfide bridges that can facilitate mutant SOD1 aggregates [262]. Sulphonylation of Cys111 in non-mutated SOD1 facilitates the enzyme adopting a Gly93Ala mutant-like 3D conformation that may interfere with fast axonal transport [263]. The S-thiolation of SOD1 protein with cysteine or GSH results in disulfide bridge formation with Cys111, changing some of the functional characteristics of the protein [259,260]. In addition, S-glutathionylation on Cys111 may cause dissociation of wild type- and familial ALS mutant G93A-SOD1 dimers, triggering monomer formation and subsequent aggregation [261]. SOD1 can be palmitoylated to different extents at Cys6, Cys111, Cys57, and Cys146, in sporadic ALS cases [264,265] (Figure 1 and Figure 2). 

The Cys6 residue may also participate in the aggregation processes and the ubiquitylation of SOD1 mutants [259]. Cys6 can be palmitoylated in native SOD1 and familial ALS SOD1 mutants in motor neuronal cells through palmitoylation on reduced disulfides [264]. Reactive cysteines are implicated in SOD1 transport into the mitochondrial intermembrane space. Indeed, SOD1 transport is mediated by its copper chaperone redox-regulation, facilitating SOD1 maturation by forming disulfide bonds that allow its retention in the mitochondrial compartment [259]. Therefore, the redox modulation of sensitive-cysteine residues in SOD1 activity plays a significant role in the pathophysiology of ALS (Figure 1 and Figure 2). However, some SOD1 mutated patients present an absence of all four cysteine residues, which argues against an explicit function of cysteine residues in aggregation mechanisms in ALS [259].

#### 7.1.2. Transactive Response DNA-Binding Protein 43 (TDP43)

TDP43 contains six cysteines, Cys173, Cys175, Cys198, and Cys244, placed in the RNA recognition domains (RRM1 and RRM2), whereas Cys39 and Cys50 stand in the N-terminal part [266]. Upon oxidative challenge, full-length TDP43 is delocalized from the nucleus to the cytosol, forming oligomers and large aggregates [267,268]. Oxidation of cysteine residues in RRM1 and RRM2 decreases protein solubility, developing intra- and intermolecular disulfide bridges and contributing to the aggregation process [259,267] (Figure 1). Oxidative stress-inducing conditions participate in forming large aggregations of proteins and oligomer-dependent oxidation of accessible cysteine residues [259,267,268], which are counteracted by reducing substances, such as high GSH levels [268]. However, a deficit of the GSH reservoir causes insolubilization and fragmentation of TDP43 in vitro [259]. The disruption of the physiological GSH/GSSG balance is crucial to activating the aggregation of mutant SOD1 and contributes to oxidizing wild-type SOD1 and TDP43, which are implicated in ALS pathophysiology through redox deregulation of the sensitive-cysteine proteome.

#### 7.1.3. Protein Disulfide Isomerases (PDIs)

ALS and other neurodegenerative diseases have shown S-nitrosylation of PDIs (the covalent acquisition of a NO molecule to a cysteine thiol group) [67,84,269,270]. In the post-mortem spinal cord from sporadic ALS and familial ALS patients, S-nitrosylated PDI concentrations are highly increased [271,272]. When S-nitrosylation affects the PDI active site, the enzymatic activity of the enzyme is inhibited, resulting in reduction in its protective functions [273] (Figure 1). PDIs are members of the thioredoxin superfamily of proteins, usually localized in the ER, catalyzing the redox modification of disulfide bridges in proteins involved in protein folding [259]. PDIA1 and PDIA3 are upregulated in spinal cords cells from sporadic ALS patients [274].

The essential function of sensitive-cysteine redox modification in ALS was confirmed through the discovery of PDI mutations in ALS patients [259]. Furthermore, PDIs cause misfolded protein inclusions in sporadic ALS patients [274], interacting with TDP43 and FUS aggregates in tissues from ALS patients [275]. The redistribution of PDIs is related to a significant increase in their enzymatic action and to decline in inactive S-nitrosylated PDI compounds [259]. PDI accumulation at the ER-mitochondria intersection can initiate apoptosis through the mitochondrial exterior membrane permeabilization pore [276]. The impaired function of PDIs in these locations was recognized in rat models of HD and AD [259], though comparable results in ALS examples have not yet been shown. These results suggest that PDI mutations and aberrant S-nitrosylation of PDIs are involved in pathophysiologic mechanisms in ALS.

#### 7.1.4. AMP-Activated Protein Kinase (AMPK)

In addition to activating the AMPK pathway by energy stress (i.e., changes in the metabolic AMP/ATP ratio), AMPK activity can be regulated by cellular redox status. AMPK is reversibly activated by the oxidation of sensitive-cysteines (Cys299/Cys303) in the AMPKα1 catalytic subunit without ATP depletion [277] (Figure 1). Similarly, redox modification of AMPK activity has been shown by indirect redox effects on mitochondrial ATP production [278]. Hydrogen peroxide can activate AMPK by oxidation and S-glutathionylation at the Cys299/Cys304 residues of its α-subunit [277] but inhibits AMPK by oxidation at the Cys130/Cys174 residues of the α-subunit, promoting its aggregation and disrupting its interaction with upstream kinases [279]. These opposite redox regulations of AMPK depend on the relative abundance of nutrients and the antioxidant capacity of cells in different physiological and pathological conditions [280,281]. Recent investigations have shown that cysteine depletion activates AMPK through calcium/calmodulin-dependent protein kinase kinase 2 (CaMKK2). Interestingly, the cysteinyl-tRNA synthetase (CARS), which plays a canonical role in protein translation, recognizes lack of cysteines and activates AMPK via the cysteine–CARS–CaMKK2–AMPKg2 system, adapting cell survival to nutrient deprivation [282]. Mitochondrial CARSs in human cells are involved in endogenous cysteine hydropersulfide (CysSSH) synthesis in vivo and even catalyze co-translational cysteine polysulfidation, which are implicated in the modulation of mitochondrial biogenesis and bioenergetics, playing a central role in redox signaling, cellular translation, and energy metabolism [283]. 

High AMPK function has been observed in motor neurons expressing SOD1 or TDP43 mutants [259,260] and in motor neuron cells of sporadic ALS and familial ALS patients [259] (Figure 1). As mentioned above, thiol groups of cysteine residues in proteins can construct covalent disulfide bonds during the oxidative folding process conferring stability and functionality to the 3D structure of proteins [284]. The stability of intra-molecular and inter-molecular disulfide links in proteins is achieved through interactions catalyzed by the PDI family of proteins in the oxidizing environment of the ER, or in proteins imported into the mitochondrial intermembrane space through the MIA pathway [259,260]. Disulfide bonds are also formed in cytosolic proteins and chaperones, such as heat shock proteins [285]. Redox-sensitive cysteines are crucial for signal transduction, transcription factor binding to DNA such as Nrf-2 and NF-kB, receptor activity, and other vital cellular functions [286].

#### 7.1.5. Fibroblast Growth Factor 2 (FGF2)

Transgenic double mouse mutants for the human SOD1G93A without the endogenous FGF-2 gene, showed a significant disease onset delay and decreased motor performance impairment compared to mutant SOD1 mice with physiological FGF-2 concentrations [287]. Moreover, the survival of the double mouse mutants was significantly prolonged for two weeks. These findings are correlated with significant preservation of the number of motoneurons and decreased astrocytosis at the end phase of the disease, suggesting a significant protective effect of FGF-2 reduction. It has been suggested that up-regulation of other neurotrophic factors, such as ciliary neurotrophic factor (CNTF) and glial-derived neurotrophic factor (GDNF), are implicated in the proven protective effects in the ALS [287]. FGF-2 has four cysteine residues of which two exposed sensitive-cysteine residues (Cys78 and Cys96) may play a role in intermolecular disulfide-bridge formation with other proteins and macromolecules [288] (Figure 1). Interestingly, FRF2 can upregulate system xc^-^ and potentially is responsible for some of its functional activities [289].

## 8. Huntington’s Disease

Huntington’s disease (HD) is an autosomal dominant condition distinguished by striatum and cerebral cortical neuronal loss. The aggregation of the mutant protein huntingtin (HTT) resulting from an expanded CAG tandem repeat (>35 repeats) in the HTT gene leads to an accumulation of the amino acid glutamine in the protein [290]. The disease pathophysiology is related to mitochondrial disturbance, oxidative stress, and excitotoxicity, derived from the protein accumulation and its interference with different cellular pathways [291,292,293].

HTT suffers numerous PTMPs, including phosphorylation, acetylation, ubiquitination, sumoylation, proteolysis, and palmitoylation, which regulate subcellular localization, protein-protein relations, folding, aggregation, and degradation, and participate in numerous cellular functions, including endocytosis, vesicle/organelle transportation and recycling, autophagy, and DNA transcription [294]. Therefore, HTT mutants disturb diverse cellular pathways contributing to disease development and progression [294].

### 8.1. Cysteinet Deregulation in HD

Most research in HD pathophysiology and progression has been focused on the deleterious effects of mutant HTT on diverse cellular pathways. Insufficient attention has though been focused on the role of the sensitive-cysteine redox proteome as a mechanism participating in the etiology and advancement of the illness [294] (Figure 1, Figure 2 and Figure 3).

#### 8.1.1. Rhes and Beclin 1

Specific striatum impairment seems to be related to the binding of mutant HTT to the striatal-selective, small G protein Rhes (Ras homolog enriched in the striatum) [295], which inhibits autophagy via Beclin-1 [296]. The Rhes protein is farnesylated at Cys263, which mediates the attachment of the protein to plasma and intracellular membranes [297,298] (Figure 1). Farnesylation is a post-translational modification consisting of the addition of a farnesyl group to proteins that facilitate protein-protein interaction and their association with membranes. Mutation of Rhes-Cys263 abolishes the sumoylation of mutant HTT and the neuropathology of HD [296]. Therefore, Rhes requires Cys263 redox regulation to maintain its physiological functionality (Figure 1). Beclin 1 is also a cysteine-rich protein that can be redox-regulated, forming part of a network that modulates autophagy and apoptosis [299]. In this network, Rubicon (RUN domain protein as Beclin 1 interacting and cysteine-rich containing) is a critical Beclin 1-binding partner, that also contains a cysteine-rich region [299] localized to the late endosome/lysosome, but it negatively regulates autophagy [299,300]. Therefore, Rhes directly interacts with HTT mutants and enhances cytotoxicity through increased sumoylation of the HTT protein, providing a potential explanation for the striatal selectivity of HD [295,301]. 

Sumoylation is a type of PTMP that participates in numerous cellular processes mediated by a small ubiquitin-like modifier (SUMO) connected to many different proteins. For example, SUMO-specific peptidase 3 (SENP3) is a particular redox-sensitive SUMO protease rapidly stabilized under oxidative stress by oxidation of its cysteine residues, blocking the ubiquitin-proteasomal degradation that occurs under physiological conditions [302] (Figure 1). Hence, ROS are necessary for autophagy initiation, while SENP3, also induced by ROS during starvation and autophagy, functions as a suppressor of autophagy [302]. DeSUMOylation of Beclin 1 restrained autophagy induction under basal conditions and starvation when SENP3 had been accumulated in response to ROS generation. Therefore, redox modification of SENP3 coupled with the deSUMOylation of Beclin 1 is critical in autophagy regulation [302].

#### 8.1.2. Cystathionine γ-Lyase (CSE)

The striatum of human HD patients shows a significant reduction (85–90%) of cystathionine γ-lyase (CSE) levels, an enzyme that forms the gasotransmitter, hydrogen sulfide (H_2_S). Further, cystathionine β-synthase (CBS) and 3-mercaptopyruvate sulfurtransferase participate in H_2_S generation, a critical gasotransmitter in redox homeostasis [301]. According to the relative vulnerability of these brain areas to HD injury, CSE depletion is selective for the striatum and the cerebral cortex. Interestingly, mutant HTT attaches and inhibits the specificity protein 1 (Sp1), a transcription factor for CSE [301]. Overexpression of Sp1 and its co-activator, TATA box binding protein (TBP)-associated factor 4 (TAF4), inverts the decreased mRNA and protein concentrations of CSE, suggesting that mutant HTT inhibits the CSE transcription factor Sp1 [301]. 

CSE can also generate cysteine from cystathionine (Figure 1). Cysteine can generate H_2_S, activating many enzymes by attaching to the thiol groups of target proteins. Sulfhydration of parkin promotes its catalytic action and appears to provide neuroprotection to the striatum in PD [303]. H_2_S sulfhydrates Keap1, a repressor of Nrf2 that drives diverse enzymes in antioxidant pathways [304]. Indeed, the beneficial effects of cysteine administration in mouse HD models may be partially mediated through H_2_S modulation [305].

#### 8.1.3. Activating Transcription Factor 4 (ATF4)

The Golgi stress response to amino acid depletion and other stress-inducing conditions acts via the PKR-like ER kinase/activating transcription factor 4 (ATF4) pathway [306,307]. One of the pathways regulated by ATF4 is the biosynthesis of cysteine through CSE up-regulation, playing a vital role in redox homeostasis (Figure 1). Thus, the involvement of the Golgi complex in neurodegenerative diseases, such as AD, ALS, and HD, can be related to deregulation of cysteine metabolism, including disturbance in the sensitive-cysteine redox proteome [306,307]. 

#### 8.1.4. Transglutaminase Cross-Linking

Brain transglutaminase activation may promote HD progression through promotion of cross-linking mutant HTT into aggregates [308,309]. The transglutaminase inhibitor cystamine is neuroprotective in transgenic mouse HD models, increasing motor performance and survival. Cystamine inhibits transglutaminase activity by promoting the oxidation of Cys370 and Cys371, two sensitive-cysteine residues on the enzyme [310] (Figure 1). In addition, cystamine can decrease HTT aggregates, inhibit caspase 3, and increase glutathione levels [309].

#### 8.1.5. Mitochondrial Antioxidant Enzymes

Mitochondrial and oxidative dysfunctions play significant roles in HD pathogenesis [311]. The antioxidant effects of cystamine have been shown in the brain of HD mouse models by increasing the concentration of cysteine [311]. The transcripts coding for proteins implicated in glutathione generation and function (gamma-glutamyl cysteine ligase, glutathione reductase), antioxidant systems (superoxide dismutase 2; SOD2), and thiol-disulfide interaction (glutaredoxin) were identified in the mouse model of HD, suggesting that the restoration of sensitive-cysteine redox homeostasis plays a critical role in neuroprotection (Figure 1 and Figure 2). SOD2 and glutaredoxin are essential enzymes for maintaining mitochondrial function, which has been shown to be disturbed in early HD [311]. Neuronal glutaredoxin is critical for enabling recovery of mitochondrial complex I activity after oxidative modification [311] (Figure 1 and Figure 2). 

Oligomers of mutant HTT can form covalent cross-linking by different actions that include noncovalent polyglutamine-dependent reactions and oxidation [312]. Indeed, cysteine oxidation at specific sites can change protein 3D structure and function by affecting oligomerization state [312]. The N-terminus of HTT has various cysteine residues placed in all but the shortest N-terminal HTT components [312]. Oxidative modifications of these residues can control HTT function and thus are essential regulators of HD pathophysiology. Similar to Cys111 in human SOD1 [312], the susceptibility of cysteine residues to oxidation depends critically on exposure of the peptide side chain to the redox environment (Figure 1 and Figure 2). The most N-terminal Cys115 residue of mammalian HTT is predisposed to oxidation because of the effects of the neighboring N-terminal histidine [312]. In addition, the N-terminal fragment of HTT, called N171, can form dimers with intermolecular disulfides. However, while the four cysteines in N171 HTT can intervene in the oligomerization, they do not act similarly. Cys115 and Cys119 are more susceptible to oligomerization than Cys137 and Cys158 [312], suggesting that cysteine redox modification participates in HD pathophysiology by changing the mutant HTT 3D structure.

#### 8.1.6. Mitochondrial Respiratory Enzymes

Regarding the mitochondrial respiratory transport chain and oxidative phosphorylation, a significant decline in mitochondrial complex IV activity and cytochrome aa3 amount has been described in HD brains [313]. Complex II-III and IV activities are also decreased in the caudate of HD persons [314,315], and supplementation with an irreversible blocker of succinate dehydrogenase (Complex II) reproduces the neurological pathology of HD [316,317]. Likewise, a transgenic mouse model of HD indicated that complex IV defect and ROS overproduction precede neuronal loss [318] (Figure 1 and Figure 2). Moreover, HTT can bind the mitochondrial enzyme GAPDH as a function of disease-related glutamine repeats [319], though the action of this enzyme was not changed in human HD brains [320]. Since the majority of these enzymatic complexes have sensitive-cysteine residues, it is suggested that a redox disturbance affects mitochondrial function in HD.

#### 8.1.7. Palmitoylation

Palmitoylation of proteins is the principal protein-lipid modification in the brain [321]. It increases the hydrophobicity of proteins by adding a palmitic acid group onto a reactive cysteine of the protein through a thioester reaction, increasing the protein trafficking, stability, membrane association, and protein-protein relationships [322]. The S-palmitoylation is a reversible process and allows the active modification of protein to occur at the synapse, playing an essential function in neuronal survival [323,324].

Cys214 is susceptible to reversible palmitoylation in HTT, a response catalyzed by two palmitoyl acyltransferases called huntingtin-interacting protein 14 and 14-like (HIP14 and HIP14L) [294,325] (Figure 1). However, some HD mouse models show that mutated HTT is less palmitoylated in the brain [325]. Using HD patient-derived lymphoblasts, it was demonstrated that mutant HTT palmitoylation was reduced with more polyQ repeats [294]. Indeed, auto-palmitoylation of HIP14 and HIP14L, and palmitoylation of numerous of their synaptic substrates, were downregulated in humanized HD mouse models and HD patient-derived lymphoblasts [294]. These findings suggest that reduced palmitoylation of mHTT in the brains of HD mouse models is not artifactual, but that aberrant palmitoylation of mutated HTT occurs in HD patients. Furthermore, it has been shown that deregulation of wild-type HTT palmitoylation in aging, combined with HTT mutations in HD patients, may be additive in disturbing palmitoylation protein levels [326].

## 9. Frontotemporal Dementia

Frontotemporal dementia (FTD) is a frequent type of illness that occurs in patients below 65 years [327] due to progressive frontotemporal lobar degeneration (FTLD) of the brain [328]. FTD is a clinical spectrum of disorders with diverse pathological and genetic backgrounds. The principal histopathology involves tau, TDP43, and FUS proteins, and mutations in several genes have been implicated [328,329,330]. About 30–50% of FTLD cases are heritable [328,329], with most disease being due to mutations within three different proteins: progranulin (encoded by GRN), tau, and a GGGGCC hexanucleotide repeat expansion in C9orf72 (chromosome 9 open reading frame 72) [330]. FTD is less typically related to mutations in TARDBP (TAR DNA binding protein, encoding TDP43) and other genes [328,329,330].

### 9.1. Cysteinet Deregulation in FTD

FTD syndromes can exist separately, or in combination with, other neurodegenerative disorders, including ALS and PD [328]. FTD develops in people with failure of one allele of the GRN gene encoding the protein progranulin, whereas loss of both alleles results in neuronal ceroid lipofuscinosis [331]. Progranulin is a full-length precursor glycoprotein formed by a signal peptide followed by 7.5 conserved tandem repeats of twelve cysteine-rich motifs processed by elastase and other proteases into six granulins (GRNs A–F) [331,332]. Progranulin and GRN act as growth factors involved in various functions, including signal transduction, inflammation, proliferation, and wound repair [331,332]. Progranulin plays a key role within neuronal lysosomes, a significant site for the production of GRNs by cysteine proteases, such as cathepsin L [333]. Specifically, as progranulin and GRNs have opposing functions in wound repair and inflammation, then conversion of progranulin to GRN by elastase acts as a molecular modulator of the host defense and wound repair [332]. Progranulin promotes neurite extension, neuronal cell survival, and differentiation [331]; it is protective in various animal models of ALS, HD, PD, and AD, independently of their association or not with TDP43 deregulation [331] (Figure 1, Figure 2 and Figure 3).

#### 9.1.1. Progranulin and Granulins

It is thought that decreased levels of progranulin cause FTD. However, the mechanism by which this protein deficiency affects neuronal function leading to neuronal death is not entirely understood. Interestingly, the pathogenic implication of two cysteine mutations (C521Y and C139R mutations) suggests that haploinsufficiency of progranulin/GRN is implicated in neurodegeneration [331,332]. These two cysteine residues are conserved among vertebrate species and located within GRN E, and GRN F. Disulfide bridges are vital for progranulin and GRN folding and structure (Figure 1). Therefore, losing one conserved cysteine will disrupt one of the disulfide bridges affecting the protein 3D conformation. It has been shown that Cys521 and Cys139 mutations affect protein mobility under oxidative conditions but not under reducing conditions [332].

These mutations affect progranulin cleavage by elastase, which is required to produce mature GRNs [331,332], suggesting that some mutations of cysteine residues disrupt the protein 3D structure affecting disulfide bond formation contributing to neuronal death (Figure 1). Therefore, in addition to low progranulin levels, the functionality of the proteins and their cleavage to generate mature GRNs are essential for normal neuronal function, and their disturbance can lead to neurodegeneration in FTD [332].

#### 9.1.2. Mitochondrial SCCPs

Mitochondria are primary cellular organelles for ATP synthesis by oxidative phosphorylation. In addition to the proteins coded on the mitochondrial genome, including 13 proteins involved in the electron transport chain and oxidative phosphorylation, around 1500 proteins encoded in the nuclear genome are delivered as precursor molecules and transported into the mitochondria by the import machinery [334]. As previously mentioned, mitochondrial impairment is associated with aging and neurodegenerative diseases caused by chronic oxidative stress emanating from ROS generation [335]. 

Some small mitochondrial proteins contain twin CX9C motifs ((CX9C)2) that participate in the transport of these proteins into the mitochondrial intermembrane space [336]. Mutations in two of these twin CX9C proteins, belonging to the mitochondrial coiled-coil-helix-coiled-coil-helix (CHCH) domain protein family, CHCHD2 and CHCHD10, have been linked to the pathogenesis of FTD, PD, ALS, and to diverse dominant inherited neurodegenerative diseases and sporadic neurodegenerative disorders [337] (Figure 1 and Figure 2). The role of the twin CX9C motifs from the CHCH domains is the building of two disulfide bridges to stabilize the helix-turn-helix fold. CHCH domains seem to hold different functions in binding to the mitochondrial import and assembly (Mia40) pathway in the IMS and proper folding of the protein. In CHCHD3, the temporary disulfide-bonded mediator with Mia40 is constructed mainly between Cys193 of CHCHD3 and the active site Cys55 in Mia40 [338]. 

Mia40 has a critical redox-active disulfide bridge in a conserved cysteine-proline-cysteine region that promotes the steady folding of the substrate by introducing disulfide bonds, thus entrapping the substrates within the IMS (Figure 1 and Figure 2). Then, Mia40 is re-oxidized by the ETS translocation variant 1 (Erv1), a protein harboring two fundamental redox-active cysteine-x-x-cysteine pairs that transport the electrons from Mia40 to flavin adenine dinucleotide (FAD) [339,340,341,342]. To achieve the disulfide interplay, Erv1 is oxidized by cytochrome c that donates the electrons through cytochrome c oxidase to oxygen in the respiratory electron chain [342].

## 10. Modulation of Cysteinet by *N*-Acetyl-Cysteine (NAC)

**Figure 4:**
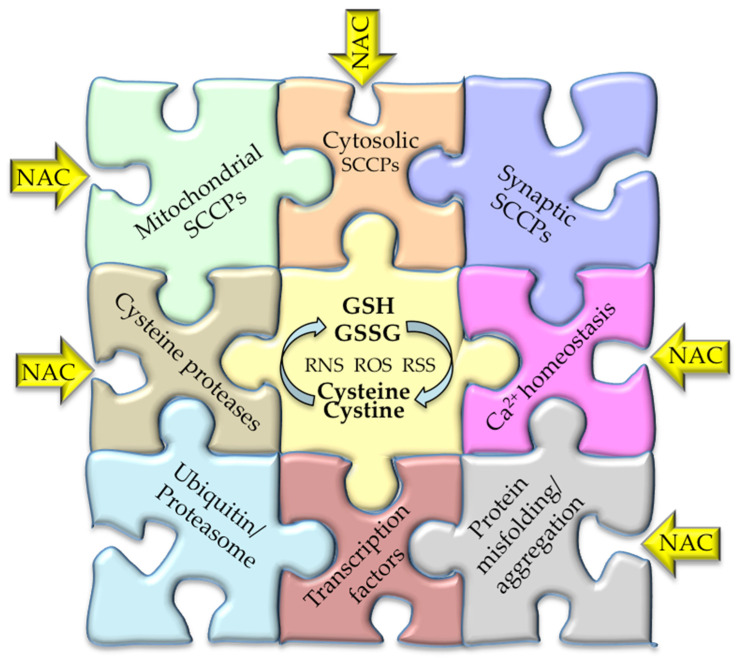
Potential role of NAC in aging and neurodegenerative disorders. Sensitive-cysteine-containing proteins (SCCPs) can suffer redox modifications of cysteine thiol (-SH) groups (S-glutathionylation, S-nitrosylation, sulfenylation, disulfide bonds formation), culminating in reversible or irreversible modification in the protein role and/or structure, resulting in cysteinet deregulation in age-associated neurodegenerative diseases. These changes can be controlled and repaired by the regular supplementation of NAC, neutralizing the harmful actions of redox changes in multiple SCCPs from diverse pathways. ROS (reactive oxygen species); RNS (reactive nitrogen species); RSS (reactive sulfur species); GSH (reduced glutathione); GSSG (oxidized glutathione); NAC (*N*-acetyl-cysteine).

Modulation of cellular redox regulation at the protein level has not explicitly been addressed. The challenge is that slight changes may affect numerous metabolic and signaling pathways in the cell and even in the extracellular medium. Any substance that can modulate the redox proteome must, therefore, be regarded as medication with significant restorative ability but potential elevated secondary consequences due to its interference with highly controlled biological mechanisms. We have proposed the probable usefulness of NAC on the redox regulation of SCCPs involved in a broad spectrum of pathways implicated in neurodegenerative and psychiatric diseases [1,2,3,4,5,29,37,187,343,344,345,346,347,348,349,350,351]. NAC is a safe pro-substrate of cysteine that can repair redox disregulated states for diverse sensitive-cysteine-bearing proteins involved in many pathways (Figure 4). GSH is a sensitive-cysteine-bearing tripeptide, which is dependent on the cysteine/cystine proportion and the redox micro-environmental balance, regulating diverse protein functions by S-glutathionylation.

NAC is a unique substance that has beneficial impacts in brain aging and neurodegenerative diseases, not only via classical antioxidant properties, but also, and even more importantly, enabling the repair and supervision of the cellular redox equilibrium through the modulation of the sensitive-cysteine network of intracellular and extracellular proteins (Figure 4) [1,2,3,4,5]. NAC is a membrane penetrable cysteine prodrug that regenerates total glutathione status and reduces excessive oxidized glutathione concentrations. Supplementation of GSH is of little value because it is oxidized in physiological conditions, having a remarkably brief half-life in human plasma (<3 min). Additionally, GSH has difficulty crossing cell membranes requiring elevated quantities to gain therapeutic concentrations [352]. On the other hand, there is adequate clinical proof that thiol-containing substances, such as NAC, can recover patients from toxic exposure to oxidative damage (e.g., acetaminophen overdose). NAC is effective in the established therapy of aged patients with obstructive pulmonary conditions, also showing significant chemo-preventive effects in lung cancer, with few harmful side effects even for longterm use [353,354]. Furthermore, NAC treatments have a healthy impact on multiple other conditions, including oncological and cardiovascular diseases, ophthalmic diseases, HIV conditions, metal toxicity, cerebral ischemic and bleeding disorders, traumatic brain damage, and even neuropsychiatric disorders [355,356,357,358].

NAC oral administration is rapidly absorbed with plasma concentrations of 16 μM and 35 μM after single doses of 600 mg/day and 1200 mg/day, respectively. The plasma half-life is estimated at 2.5 h, and no NAC is discernible 10–12 h following oral administration [359]. The terminal half-life of reduced NAC after oral administration is 6.25 h. It is quickly degraded and integrated into proteins with low concentrations of oxidized NAC detectable after several hours [345,346]. Therefore, NAC is employed routinely in clinical practice, traveling across the blood-brain barrier (BBB), and immediately interacting with essential SCCPs in the brain, compensating brain-related aging and age-associated neurodegeneration [1,2,3,4,5]. NAC can likely repair redox imbalance by replenishing mitochondrial soluble and protein-linked thiols, restoring mitochondrial bio-energetic capability and adequate ROS concentrations, and decreasing the oxidative damage associated with brain aging. 

### 10.1. NAC in Brain Aging and Neurodegenerative Diseases

Considering brain aging as the result of a progressive decline in the bio-energetic ability of neural cells accompanied by deregulation of metabolic homeostasis, involving the redox balance and proteostasis, no therapeutic or preventive interventions have achieved significant effects. Brain aging seems to depend on the inability of the mitochondria to support the bio-energetic capacity, which is mediated by the redox disturbance of the mitochondrial structure, biogenesis, and physiology associated with ROS overproduction [360,361,362,363,364,365,366]. Age-associated decline in the activity of various mitochondrial ROS-scavenging enzymes and the proliferation of mutations in mitochondrial DNA (mtDNA) may also influence cellular stem compartment. Thus, a vicious cycle may develop because of somatic mtDNA mutations, impaired mitochondrial respiratory chain activity, and oxidative phosphorylation deficiency, resulting in further ROS generation and accumulation of damaged proteins, lipids, and DNA [363]. Therefore, mitochondria play a crucial function in starting and driving the oxidative stress that causes the evolution of brain aging.

Numerous approaches have been used to neutralize the detrimental effects of brain aging. In particular, antioxidant molecules and dietary complements can potentially improve age-associated depletion of the bio-energetic ability of cells [367,368]. The beneficial effects of caloric restriction in increasing neuron resistance to age-related disease has been demonstrated [367,369]. Other approaches include anti-apoptotic agents targeting essential cellular proteins [370,371], while statins seem to prevent brain aging and age-associated neurodegenerative illness through different mechanisms (Figure 4) [372].

Modification of the redox state of critical functional cysteines in proteins by NAC administration has not been systematically examined previously. Redox modulation of SCCPs may illustrate numerous results of NAC treatment, including amelioration of metabolic processes, improvement of the immune system, and anti-aging effects [1,2,3,4,5,373]. For example, the addition of NAC influenced the initiation and reduced the severity of early aging in Bmal1-deficient mice. Bmal1 is a circadian clock protein implicated in tissue homeostasis by the direct regulation of ROS, acting as a transcription factor of critical components of the circadian clock. NAC attenuated the development of the age-related phenotype of Bmal1−/− mice decreasing the development of cataracts and extending the animals’ lifespan [374]. Studies in synaptic mitochondria from aged mice treated with NAC showed its anti-aging properties, increasing ATP levels through the activation of the mitochondrial complexes of the respiratory chain and oxidative phosphorylation, restoring GSH levels, and decreasing lipid and protein oxidation in presynaptic terminals [343,344,345,346,347,365].

Another beneficial action of NAC in brain aging is associated with glutamate uptake by astrocytes and neuron cells expressing the excitatory amino acid carrier-1 which can even transport the cysteine required for GSH biosynthesis. NAC reversed the GSH depletion and associated oxidative damage in a deficient mouse model of these carriers, suggesting that the excitatory amino-acid carrier-1 may be necessary for cysteine import and GSH biosynthesis in neuronal cells [375]. NAC can enhance the physiological function of mitochondrial complexes I, IV, and V in the synaptic mitochondria of old mice, likely restoring the oxidative damage of sensitive-cysteine residues in these proteins [343,344,345,346,347]. In vivo experiments confirmed that these enzymatic functions were restored by regular NAC supplementation, with rising ATP and GSH concentrations, and reduction in lipid and protein oxidative damage in presynaptic terminals (Figure 4) [350,351].

Redox equilibrium is the primary process through which reactive species (ROS, RNS, and RSS) integrate the regulation of intracellular metabolic routes, principally mediated by SCCPs [1]. Reactive species also modulate transcription factors, such as nuclear factor kappa B (NF-ĸB), activator protein 1 (AP-1), and the inhibitor of nuclear factor-kappa B kinase (IKK), all of which contain redox-sensitive cysteine residues [376]. Specifically, NF-ĸB contains two redox-sensitive cysteine residues (Cys38 and Cys62) that are essential for its function [377,378], IKK contains Cys179 that participates in its catalytic kinase activity [379], and the transcription factor AP-1 binds DNA under the control of redox-sensitive cysteines [380]. In this context, NAC can directly regulate common transcription factors both in vitro and in vivo [355]. NAC can suppress NF-kB in oxidative stress and clinical sepsis, diminishing the next cytokine generation [355,381]. NF-kB is physiologically attached to its inhibitor (I-kB), preventing its nuclear transport. Dissociation of I-kB after its phosphorylation by IKK allows NF-kB to transport into the cellular nucleus [355]. Furthermore, NAC inhibits APP gene transcription in neuroblastoma cells by reducing the crucial activity of NF-kB (Figure 4) [382]. These NAC actions are likely produced by their capacity to manage the reactive cysteines of the cysteine redox proteome [1,2,3,4,5].

A potential restorative activity of NAC therapy is the inhibition of age-associated protein oxidation, misfolding, and aggregation by preventing sensitive-cysteine oxidative impairment related to aging. The 3D structures of proteins can suffer conformational changes when they accumulate oxidative damage [383]. The shift from a-helix to b-sheet is typical of amyloid and other protein accumulations. Structural modifications probably occur in proteins with redundant amino acid arrangements, such as polyglutamine in HD. Chaperones assist proteins in reaching their functional structure in physiological situations. However, in aging, the delicate equilibrium among protein synthesis, folding, and clearance can diminish, culminating in the aggregation of misfolded proteins. The assemblage of misfolded proteins contributes to the pathogenesis of age-associated neurodegenerative diseases, such as AD, PD, and HD, which NAC treatment can presumably repair. Cysteine-bearing compounds, such as GSH, NAC, and *N*-acetyl-cysteine amide (NACA) may preclude exosome production from oxidative and pro-inflammatory triggers through scavenging and preventing thiol-reactive substrates [195,196]. NAC can correct exosome induction, arrangements, and actions to comparable levels of unexposed cells instead of completely inhibiting exosome signaling. Hence, NAC prevents oxidative shifts in exosome signaling without interrupting their physiological roles [195,196].

Cysteine impedes the aggregation of Aβ1-40 and Aβ1-42 and the accumulation of amyloidogenic peptides, and it is less cytotoxic than catechin, the most precise blocker of amyloid fibril accumulation [384]. Moreover, the beneficial impact of NAC was confirmed in a mouse HD model [305]. Therefore, NAC administration can partially restore the age-associated accumulation of misfolded proteins, neutralizing one of the processes contributing to neurodegenerative disorder development (Figure 4).

NAC may defend neurons via antioxidant properties and by controlling the redox standing of sensitive-cysteines in numerous proteins, thereby rejuvenating critical cellular pathways that support neural cell survival (Figure 4) [1,2,3,4,5,187]. NAC can activate the Ras-ERK (extracellular signal-regulated kinase) pathway in vitro through non-antioxidant mechanisms, protecting neuronal cells from death in the absence of trophic factors. Since Ras proteins contain essential reactive cysteines, it was suggested that NAC could trigger Ras by its reducing capability [385,386]. Some analyses showed that NAC could defend human neurons from the cerebral cortex against death induced by Aβ-amyloid 1−42 [187], causing p35/Cdk5 activation and decreasing phosphorylation/deactivation of the MLK3-MKK7-JNK3 signaling pathway [reviewed in 1]. Cdk5 is a cyclin-dependent kinase triggered via p35, p25, and p39 [387,388,389], acting as a neuronal-specific kinase implicated in cell preservation, axonal guidance, neuronal migration, and modulation of synaptic spine density [389]. Deregulation of Cdk5 function mediates the pathophysiology of diverse neurodegenerative illnesses, such as AD, ALS, PD, and HD [390]. Elevated Cdk5 activation by proteolytic cleavage of p35 to p25, through the calcium-activated protease calpain, can contribute to neurotoxicity. S-nitrosylation of Cys83 and Cys157 activates Cdk5 forming SNO-Cdk5, contributing to amyloid-β (Aβ) peptide-induced dendritic spine loss [390,391]. Similarly, increased concentrations of SNO-Cdk5 have been observed in postmortem AD brains compared to control human brains, suggesting that S-nitrosylation of Cdk5 disturbs its enzymatic activity contributing to AD pathophysiology [390]. On the other hand, mixed lineage kinase 3 (MLK3) can be activated under ischemic stress. Its S-nitrosylation at the sensitive Cys688 residue contributes to its dimerization and activation, which has been implicated in brain ischemia/reperfusion damage [392]. However, NAC can inhibit the activation increase in MLK3 in the earlier phases of ischemia/reperfusion, indicating that MLK3 activation is again associated with ROS-mediated events following brain hypoxia [393]. Interestingly, MLK3 activation can phosphorylate other SCCPs, such as Pin1 [394], increasing their catalytic activity and nuclear translocation [395].

Finally, since the oxidative changes of sensitive-cysteines on proteins can influence exosome building and functions, thiol-protecting compounds, such as NAC, may protect against harmful disruption of extracellular vesicle mechanisms under pro-oxidant situations. NAC supplementation may repair vesicular physiology instead of impeding exosome processes [195], repairing extracellular vesicle signaling by modulating the functional cysteine proteome. Hence, a probable restorative action of NAC supplementation in neurodegenerative diseases is the redox restoration of diverse structural, enzymatic, and signaling proteins implicated in extracellular vesicle formation and signaling. This contemporary notion reinforces the usefulness of NAC as a prophylactic and rejuvenating compound against brain aging and neurodegeneration, founded on the suggested restoration of the cysteine proteome [1,2,3].

#### 10.1.1. Preclinical and Clinical Studies of NAC in AD

NAC has been investigated in AD mice models [396,397]. Nevertheless, studies in humans with neurodegenerative disorders are insufficient. Preclinical examinations have shown that NAC supplementation is helpful in AD murine models neutralizing oxidative impairment [396,397,398] and diminishing Aβ1-40 and Aβ1-42 concentrations [399]. Similarly, NAC can ameliorate the behavior of animals in the T-maze foot-shock escape procedure [400]. NAC supplementation to human APP/PS-1 knock-in mice preceding brain accumulation of Aβ, diminished protein and lipid oxidative damage, protein nitration, and raised glutathione peroxidase and reductase activities compared to normal aged animals [401]. Regular NAC therapy in human double mutant APP/PS-1 knock-in mice showed little gain in peptidyl-prolyl isomerase 1 (Pin1) amounts, likely reducing Aβ generated oxidative damage [401]. Pin1 has been implicated in AD [402] by oxidative modification in human AD brains [394]. The oxidation of Cys113 blocks Pin1 catalytic activity, and the substitution of Cys113 inactivates the Pin1 ability to isomerize with tau protein. Since Cys113-oxidized Pin1 is remarkably raised in the human AD brain compared to control aged people [394], these results show another redox-disturbed critical sensitive-cysteine residue in AD that can be regulated by NAC supplementation. Likewise, in vitro studies with NAC in cultured neuroblastoma cells affected APP metabolism by modifying β-secretase and γ-secretase actions and reduced phosphorylated tau concentrations in the absence of stressful conditions [403].

AD postmortem cerebral cortex exhibited a substantial reduction in mitochondrial cytochrome c oxidase activity (mitochondrial complex IV). Moreover, cybrid cells transferred with mitochondria from AD platelets displayed complex IV deficiencies and increased ROS generation [404]. Accordingly, AD patients have mitochondrial DNA mutations [405,406,407], indicating effects on mitochondrial DNA impairment and subsequent injury of neuronal bioenergetic ability [408]. Some investigations showed a reduction in mitochondrial mRNA encoding complex IV in AD patients’ temporal cortex and hippocampus [409,410]. In this regard, in vivo chronic oral NAC supplementation restored complex IV activity in synaptic mitochondria from old mice, decreasing mitochondrial lipids and oxidative protein damage [343,344,346]. This impact of NAC treatment correlated with a decrease in age-related memory deterioration in aged mice [350]. An exciting study investigated the effect of lipoic acid and NAC on fibroblasts obtained from people with AD, age-matched and young controls. Fibroblasts from AD patients displayed the most elevated amounts of oxidative damage, and both compounds diminished oxidative stress, apoptotic markers, and mitochondrial dysfunction compared to controls, suggesting that mitochondria could be a critical target of NAC supplementation [411].

NAC supplementation (50 mg/kg/day in three divided daily doses) for 24 weeks in probable AD patients caused a beneficial trend on nearly every outcome measured, including the cystine-glutamate antiporter system, and marked progress in some cognitive tasks [412]. The cystine-glutamate antiporter system uptakes cystine, exerting beneficial effects, such as reducing β-amyloid stress, preventing oxidative-induced apoptosis, and improving sodium-dependent glutamate transporter activity [403,413]. Another clinical study with moderate to late-stage AD patients improved neuropsychiatric tests following chronic NAC administration in addition to folic acid, vitamin B12, α-tocopherol, S-adenosyl methionine, and acetyl-L-carnitine [414]. Consistent with this, a patient with potential AD and hyperhomocysteinemia exhibited substantial clinical amelioration following NAC, vitamin B12, and folic acid administration [415].

NAC can improve metabolic function, calcium signaling, protein misfolding, and proteostasis, all of which are implicated in AD progression. Indeed, NAC modified the oxidative damage of plasma proteins, such as transthyretin (TTR) by dose-dependent interaction with the reactive cysteine of the protein [416,417]. TTR blocks the expression of AD phenotype in transgenic animal models, reducing cerebral Aβ deposition [418]. 

A potential NAC action is the modulation of gene transcription and expression signals depending on the redox sensitivity of cysteine residues in the transcription factors themself. NAC can effectively modulate key transcription factors in vitro and in vivo [146]. Previous treatment of human neuroblastoma cells with NAC diminished β-amyloidogenesis induced by two oxysterols suggesting that this antioxidant can protect against cholesterol oxidation products modulating APP and β-secretase activity in the brain [419].

#### 10.1.2. Preclinical and Clinical Studies of NAC in PD

Regarding the role of NAC in PD, a recent investigation showed that oxidative stressinducing conditions and toxic α-synuclein conditions resulted in c-Abl activation. NAC administration reversed this effect, accompanied by improvement in dopaminergic neuronal death and motor amelioration in a murine PD model [420]. Accordingly, recently a notable gain in DAT binding has been reported in the caudate and putamen of patients with idiopathic PD supplemented with NAC therapy. The investigation revealed substantial improvement in the dopaminergic system function estimated by DaTscan SPECT imaging and amelioration in the symptomatology of the disease [421,422]. Moreover, in experimental PD models, peroxiredoxin 1 (Prx1) has also been implicated in dopaminergic neurodegeneration. NAC blocked the drug-induced oxidative damage of peroxiredoxins, suggesting that functional cysteines are critical in the susceptibility of peroxiredoxins to oxidative damage [423]. Therefore, complementing the known actions of NAC as a free radical scavenger and its ability to replenish GSH levels, it also can modify the thiolic groups of sensitive-cysteines in diverse proteins implicated in PD pathophysiology contributing to α-synuclein disruption [4,5].

The principal excitatory neurotransmitter in the mammalian CNS is the amino acid glutamate. Glutamate is removed from the synaptic space by the excitatory amino acid transporter (EAAT) lineage, formed of five components. EAAT1 and EAAT2 are expressed predominantly in glia, whereas EAAT3, EAAT4, and EAAT5 are mainly represented in neurons [424]. Dysfunction of these EAATs results in excitotoxicity associated with neurological conditions, such as ischemia, ALS, AD, and epilepsy [424]. EAAT3 also transports cysteine with higher affinity than other transporter family members [425], and has been implicated in maintaining intracellular redox potential [426]. Indeed, EAAT3 deficient mice showed lowered concentrations of neuronal glutathione, augmented oxidative damage, and increased neuronal death in the dopaminergic neurons of the substantia nigra during aging [427]. Treatment of these mice with NAC, which was taken up by cells without the participation of EAAT3, rescued the phenotype, reinforcing the notion that EAAT3 activity is essential for the cysteine redox balance [426,427]. The particular high-affinity and transportation of acidic amino acids by EAATs concerns the positively charged residue in the EAAT3, which is preserved in all EAATs [426]. The replacement of Arg447 by cysteine in EAAT3 transforms the protein from an acidic amino acid carrier to one that carries neutral amino acids [426].

#### 10.1.3. Preclinical and Clinical Studies of NAC in HD

NAC prevented mitochondrial dysfunction in a rat HD model [428]. Injection of rats with 3-nitropropionic acid (3-NP) induced an irreversible inhibition of mitochondrial complex II, resulting in oxidative damage in both striatum and cortical synaptosomes of treated animals [429]. Pretreatment with NAC starting two hours before 3-NP injection rescued the animals against oxidative injury resulting in a significant reduction in striatal lesions [429]. In addition, ROS overproduction and lipid peroxidation in the mitochondria of 3-NP-injected animals was associated with decreased thiol levels and SOD activity in mitochondria in rats not treated with NAC. Nevertheless, NAC treatment could reverse 3-NP-induced mitochondrial disturbances and behavioral deficiencies [428,429], indicating a potential therapeutic impact of NAC in this HD model, likely mediated by a restoration of the sensitive-cysteine redox proteome. Moreover, chronic NAC administration reduced the onset and progression of motor deficits in a transgenic mouse model of HD [305]. These transgenic mice showed reduced mitochondrial respiratory ability in the striatum, which was rescued by NAC treatment associated with ameliorating oxidative damage in mitochondria [305]. 

Likewise, NAC increased the glutamate neurotransmitter in HD mice in a glutamate transporter-dependent way [430]. However, the glutathione redox system was unchanged, suggesting that NAC may act by independent antioxidant mechanisms [430], contributing to in vivo modifications in glutamate transporter proteins in HD mice and HD patients. We propose that supplementing diets with NAC can compensate for low levels of cysteine, reducing oxidative stress and restoring, at least partially, the enzymatic activity of sensitive-cysteine proteins, such as CSE in HD, which in turn generate H_2_S, cysteine, and other thiol products, such as cysteamine and CoA.

#### 10.1.4. Preclinical and Clinical Studies of NAC in ALS

NAC administration can reduce oxidative damage and mitochondrial dysregulation in human neuroblastoma cells (SH-SY5Y) with the G93A-SOD1 mutation, and delay the beginning of motor injuries and enhance survival in G93A-SOD1 mutated mice [431,432]. NAC treatment of patients with ALS partially modified the course of the disease [433]. A randomized, double-blind, and controlled trial showed that NAC produced a moderate, statistically non-significant gain in survival without proof of a decline in disease advancement. The amount employed was much lower than the dose utilized in clinical trials with beneficial effects for other CNS disorders [434]. Likewise, the impact of NAC may rely on the variety of ALS expressed since NAC was able to enhance survival in subgroups of patients with limb onset illness compared to those with bulbar onset [433].

## 11. Conclusions and Future Perspectives

Future studies will be necessary regarding the essential role of sensitive-cysteine residues in maintaining protein structures and functions and the implications of NAC for diverse clinical and empirical conditions. Here, we emphasize the uniqueness of NAC as the best compound that is presently extensively employed in clinical routine, that can traverse the blood-brain barrier (BBB), and can potentially modulate key SCCPs in the brain, counteracting significant harmful effects of brain aging and associated neurodegeneration [435]. Current studies have revealed the beneficial influence of NAC on the dopamine system related to improving clinical outcomes in PD [421,422]. In these studies, DaTScans before and after therapy with NAC over 90 days measured dopamine transporter (DAT) binding. The results demonstrated a notable improvement in caudate and putamen DAT binding in PD patients supplemented with NAC compared to the control group. Additionally, clinical manifestations, estimated through the unified Parkinson’s disease rating scale score, were remarkably enhanced in the NAC patients [421,422]. 

Determining cysteinet deregulation in clinical samples from complex neurodegenerative disorders is technically challenging. However, a recent prospective study has shown that plasma GSH concentrations and mental function decreased after two years in patients with mild cognitive impairment (MCI) [436]. The redox status of human serum albumin, the major protein in the blood, seems to play a predominant role in redox regulation through its thiol group in the redox-sensitive Cys34 [437]. Moreover, it can serve as a prognostic biomarker of oxidative damage in chronic degenerative diseases [437,438]. Cys34 in albumin exists in equilibrium between reduced and oxidized forms, and their ratio, in addition to GSH levels, may indicate the progression of oxidative damage in complex degenerative diseases [438]. For example, albumin in oxidized form is about 35% under healthy physiological conditions, but it can rise to 70% after oxidative insults [439]. Interestingly, albumin Cys34 redox modification in vivo is mainly due to the interaction with the cysteine/cysteine ratio [439], and therefore with GSH/GSSG ratio without the involvement of any enzymatic support. An analogous mechanism is conceivable in many SCCPs under physiological and pathological conditions. Thus, differential cysteine labeling investigations and global label-free proteomics analyses in blood, CSF, and brain samples seem to be necessary to comprehend the function of the cysteinet disturbance in aging and age-related neurodegenerative disorders (Figure 3).

The number of SCCPs implicated in different neurodegenerative diseases is continuously increasing, and their participation in each specific disorder needs to be investigated without delay. For example, the involvement of APOE4 and tau variability in the development of dementia in PD has recently been recognized [440]. Additionally, the role of oxidative damage and disulfide bond formation in tau [441] and other SCCPs are critical triggering mechanisms leading to neurodegenerative disorders and pathogenic progression factors. Therefore, NAC may offer an effective therapeutic strategy to decrease the generation and spread of pathological proteins [442].

We propose large-scale investigations in selected groups of people over 40 years of age with decreased blood GSH levels, comorbidities, and/or MCI by supplementing the diet with low doses (between 1800–3000 mg/week) of NAC, a promising and well-tolerated therapeutic agent suitable for long-term use. Although MCI has been designed to recognize a prodromal stage of dementia, identifying cognitive impairment in the preclinical or earliest clinical stages is not easy. Clinical trials aiming to decrease oxidative injury remain unsuccessful for age-associated neurodegenerative diseases, and GSH scavengers can even impair the physiological function of ROS, RNS, and RSS. However, early intervention is essential for preventing the evolution of age-related neurodegeneration because pathological brain damages have been found years or even decades before the cognitive decline was clinically evident. Therefore, we propose an early intervention using low doses of NAC supplementation in people over 40 years of age with comorbidities (e.g., diabetes, hypertension, obesity, and cardiovascular disturbances) or presenting disruption in GSH and albumin redox ratios. NAC can likely maintain and repair redox homeostasis dysregulation by replenishing free (cysteine/glutathione) and protein-linked thiols to restore mitochondrial bio-energetic ability and biogenesis. NAC can be regarded as an effective compound due to its capacity to regulate a diversity of vital proteins from many cellular processes. Consequently, its doses must be accurately investigated. Much work remains to be done to examine the redox modulation implicated in the principal pathways in neural cells and its role in degenerative disorders. However, NAC can play an essential role in preventing age-associated oxidative dysregulation in the brain.
